# ARTNet: Adaptive channel-wise and Region-aware Transformer Network for diabetic retinopathy segmentation and classification

**DOI:** 10.3389/frai.2026.1913611

**Published:** 2026-08-26

**Authors:** Annuj Kumar, Munjam Shruthi, Thiruppathy Sujeeth, Abubacker Kaja Mohideen, Aravindkumar Sekar

**Affiliations:** School of Computer Science and Engineering, Vellore Institute of Technology, Chennai, India

**Keywords:** attention mechanism, diabetic retinopathy, feature fusion, retinal lesion detection, retinal lesion segmentation

## Abstract

Early and accurate detection of diabetic retinopathy (DR) is essential to prevent irreversible vision loss; however, manual screening is labor-intensive and subject to inter-observer variability. To address these limitations, we propose ARTNet, an Adaptive channel-wise and Region-aware Transformer Network for automated DR classification and segmentation from retinal fundus images. ARTNet integrates three sub-network mechanisms. The Adaptive Channel-wise Feature Network (ACFNet) performs channel recalibration using dual pooling and shared multilayer perceptrons to enhance discriminative retinal representations while suppressing irrelevant responses. The Ophthalmic Region-Aware Attention Network (ORAANet) applies spatial attention to highlight clinically significant regions, including lesions and abnormal vasculature. The Retinal Patch Aggregation Encoder Network (RPAENet), built on multi-head self-attention, captures long-range dependencies and global retinal context for hierarchical feature modeling. Convolutional refinement and global average pooling enable robust five-class DR classification, while a class-balanced focal loss mitigates data imbalance and improves minority-class sensitivity. Extensive experiments on benchmark datasets demonstrate the superiority of ARTNet over intermediate and state-of-the-art models. On the Diabetic Retinopathy Detection dataset, ARTNet achieves 96.45% accuracy, 96.85% precision, 96.34% recall, and 96.60% F1-score. The model is further validated on the APTOS-2019 Blindness Detection and IDRiD datasets. Classification performance is evaluated using image-level DR grading metrics, whereas lesion segmentation performance is evaluated using the pixel-level lesion annotations available only in the IDRiD dataset. Results show that dual attention with transformer-based global reasoning improves feature representation and classification reliability. Its efficiency and stability support real-time ophthalmic screening and a clinical decision system.

## 1 Introduction

Diabetes mellitus has emerged as a worldwide healthcare crisis, whose increasing prevalence poses serious challenges to healthcare systems globally (Saeedi et al., 2019). One of the main microvascular complications of diabetes is Diabetic Retinopathy (DR), a condition that is progressive in nature and has evolved into the leading cause of visual impairment and preventable blindness in working-age adults (Li J.-Q. et al., 2020; An et al., 2019). Clinical evidence is almost unanimous that early, frequent screening is capable of effectively stopping the advance of DR and preventing advanced vision loss (Jones and Edwards, 2010). Yet the present gold standard in screening depends on human interpretation by ophthalmologists of retinal fundus images. The process is time-consuming, subjective, and reliant on highly trained experts available at a given moment, and it presents a major bottleneck that restricts access to care for the enormous and increasing diabetic population (Sebastian et al., 2023; Khan M. B. et al., 2023). To address these constraints, the establishment of automated systems for retinal image analysis has emerged as an important research area and a public health strategic priority (Pratt et al., 2016).

The diagnosis of DR relies on the recognition of certain pathological lesions, such as microaneurysms (MAs), hemorrhages (HEs), and hard exudates (EXs) (Ali Shah et al., 2016). Automating their detection is a challenging task as they have low contrast and a highly variable appearance. For example, microaneurysms, usually the initial clinical feature, are very small and have low contrast so that they appear barely visible against the retinal background (Dai et al., 2019). In addition, the behavior of computer systems is frequently made difficult by inconsistencies in image quality, including bad light and artifacts, typical in real clinical environments (Xu et al., 2023).

Early CAD systems employed conventional machine learning methods that relied on handcrafted feature engineering—a laborious process in which algorithms were designed specifically to measure lesion features such as color, shape, and texture (Almattar et al., 2023). Although groundbreaking, these approaches tended to be brittle and could not easily generalize to varied patient populations and imaging hardware (Sebastian et al., 2023). The emergence of deep learning, notably Convolutional Neural Networks (CNNs), has seen a paradigm shift. Through the automatic learning of a hierarchy of relevant features from image data, deep learning models have long shown performance comparable to, and in some cases better than, that of human experts in DR detection (Gargeya and Leng, 2017; Ting et al., 2019).

While CNNs excel at local feature extraction, their inherent architecture, which relies on local receptive fields, limits their ability to model long-range spatial dependencies within an image (Wang et al., 2021). This is a notable drawback in ophthalmology, where the spatial relationship between distant lesions can be clinically significant. Here, Vision Transformers (ViTs) have gained significant attention. Using self-attention mechanisms helps to model global relationships across the entire image. Therefore, ViTs are much stronger in understanding complex contextual information. However, ViTs are not without their limitations; typically requiring vast amounts of training data, ViTs have a weaker inductive bias compared to CNNs, which becomes a challenge when dealing with limited or specialized medical datasets. This has inspired research into hybrid architectures that try to combine the strengths of CNNs in local feature extraction with the global context modeling ability of ViTs. In tandem with architectural innovation, data-centric strategies have become crucial to building robust and generalizable models. Specifically, one of the most popular strategies to alleviate domain shift relies on multi-source datasets, where models are trained on a diverse collection of images from different populations, clinics, and camera systems. Publicly available datasets like EyePACS, Messidor, and IDRiD offer a rich, varied source of data for these purposes. Advanced image pre-processing also plays a vital role. Methods such as Contrast Limited Adaptive Histogram Equalization are often used to normalize images and make subtle lesions more visible, ensuring that the model receives high-quality, standardized input irrespective of the original condition of the image.

Despite these advances, the translation of deep learning models into reliable clinical tools is impeded by several critical limitations. These define the primary research gap that this study tries to address: Domain Shift: The key challenge is the inability of models to generalize. The performance may be poor and limited in real-world applications and scalability when a model trained on data from one hospital or one camera system is applied to data from another (Shamshad et al., 2023; Dai et al., 2021). Lack of Robustness: Real clinical practice inevitably produces images with variable quality. The diagnostic accuracy of many models degrades significantly in the presence of artifacts, poor focus, and other image imperfections (Qian et al., 2024). Interpretability Issues: Most high-performing deep learning models operate like “black boxes,” where their decision-making process is not transparent. This lack of transparency is one of the most critical challenges to clinical adoption, since it prevents clinicians from trusting the model's output.

The most significant research gap lies in the development of a unified framework. Such a framework should overcome the limitations of existing approaches. It must deliver high diagnostic accuracy while maintaining robustness across diverse datasets. In addition, the model should demonstrate strong generalization capability under varying imaging conditions. To address these challenges, a novel Adaptive channel-wise and Region-aware Transformer Network (ARTNet) architecture is proposed for the automated detection and segmentation of Diabetic Retinopathy (DR). The primary objective of this study is to design and develop an advanced DR detection network that strategically integrates Adaptive Channel-Wise Attention, Region-Aware Attention, and a Retinal Patch Aggregation Encoder Network (RPAENet) to enhance diagnostic accuracy and reliability. By combining these complementary mechanisms, ARTNet aims to strengthen discriminative feature learning, improve lesion localization, and effectively capture both local pathological details and global contextual dependencies within retinal images.

The main contributions of our study are summarized as follows:

- A novel Adaptive channel-wise and Region-aware Transformer Network (ARTNet) is proposed for diabetic retinopathy analysis. It integrates channel adaptation, region-aware attention, and transformer-based global modeling to capture fine-grained and contextual retinal features.
- Adaptive Channel-wise Feature Network, which employs dual pooling–driven channel reweighting to enhance diagnostically relevant lesion features. It also suppresses redundant background responses.
- Ophthalmic Region-Aware Attention Network is designed to localize clinically significant retinal regions through region-aware attention learning. This improves lesion localization and enhances model interpretability.
- Retinal Patch Aggregation Encoder Network (RPAENet), combining multi-head self-attention and convolutional refinement to model long-range dependencies and preserve spatial hierarchies for robust grading and segmentation.
- Extensive experiments were conducted separately for DR grading (classification) and lesion segmentation. Classification was evaluated on the Diabetic Retinopathy Detection, APTOS-2019, and IDRiD datasets, whereas lesion segmentation was evaluated only on datasets providing pixel-level lesion annotations.

The organization of this article is as follows: Relevant research on diabetic retinopathy detection, segmentation, and classification from retinal fundus images is reviewed in Section 2. The proposed ARTNet architecture, incorporating adaptive channel modeling, region-aware attention, and the Retinal Patch Aggregation Encoder Network, is detailed in Section 3. Section 4 describes the experimental setup, datasets, and implementation details. Results and performance analysis of the proposed model are presented in Section 5. Finally, the conclusion is summarized in Section 7.

## 2 Related works

In this section, we analyzed various recent and related research articles that address various approaches developed for diabetic retinopathy segmentation and classification.

### 2.1 Deep learning and generative adversarial network-based retinopathy segmentation

A Convolutional Neural Network (CNN) based model is introduced in Ali et al. (2023) for detecting and classifying Diabetic Retinopathy (DR) by integrating two different features extracted from InceptionV3 and Resnet50 models (IR-CNN). They trained their model using the Optical Coherence Tomography (OCT) dataset, which consists of 44,119 color fundus images, and also classified them into five different types: mild, moderate, severe, non-diabetic retinopathy, and proliferative diabetic retinopathy. TMM-Nets (Liu et al., 2023) proposed a cycle generative adversarial network-based model for generating Fundus Photography (FP) to Fundus Fluorescence Angiography (FFA) images. TMM-Nets also help to identify the data at several scales, with a lesion region that supports the attention model to detect the lesion regions more accurately.

Patho-GAN is introduced in Niu et al. (2022) to synthesize retinal images. They also incorporated the pathological descriptions for generating the diabetic retinopathy images. Additionally, perceptual and severity loss functions are used along with the adversarial loss function. Frechet inception distance score and Mean squared error are used as the evaluation metric to measure the performance of Patho-GAN. An iterative algorithm is implemented to detect and classify the retinal fundus images (Narasimha-Iyer et al., 2006). They generated the color-coded output to highlight the diabetic retinopathy-infected region using a Bayesian detection algorithm.

Class-Imbalanced Learning network (Xie et al., 2023) is implemented using GAN. It is developed to generate the retinal images based on conditional information. Adversarial and classification loss functions are incorporated to generate more appropriate and realistic retinal images. A lightweight W-capsule network is introduced in Balla et al. (2024), for segmenting the color region of the fundus images more efficiently. A dual stream segmentation network (Xiang et al., 2023) is implemented to segment the retinal lesion region, which is also associated with the conditional GAN model. In the encoding part, a multi-level fuse block is deployed for extracting features, and in the decoder part, a semi-supervised adversarial network is used for segmentation.

Vij and Arora (2024) designed a hybrid evolutionary weighted ensemble upon the deep transfer learning, which helps to detect diabetic retinopathy. They have employed three different deep transfer learning models, which include ResNet 34, Inception V3, and VGG16. They used an improved U-Net architecture for semantic segmentation. Their model was trained using an ImageNet-pretrained model with an accuracy of 0.9571. When comparing the results, the maximum accuracy was achieved in their proposed approach of 0.9960. A deep multi-scale framework is emphasized in Guo T. et al. (2024) for segmenting the diabetic retinopathy lesions. The primary focus of the research work is the formulation of a model using the CNN for segmenting 4 different lesions in retinopathy images, which include Microaneurysm, Hemorrhage, hard and soft exudates. In the first stage, an FCN is created and trained for segmentation, and in the second stage, a CNN is trained to fuse the N predictions of the FCN. The input layer is sequentially connected from a convolution layer, a BN layer, a Relu layer, and then to an MP layer.

In Alharbi and Gupta (2023), a deep feature fused residual network along with the U-Net is implemented to segment diabetic retinopathy images. The primary focus of the research work is to segment the lesions more specifically, hemorrhage, hard exudates, and the optic disc of the fundus image. They achieve the accuracy score of 98% for hemorrhage, 99% for hard exudates, and 99% for optic disc. In order to fully automate the lesion segmentation from retinal images, RILBY-Ynet (Geetha Pavani et al., 2023) provides a promising result for diagnosing diabetic retinopathy. Residual Inception Local Binary Pattern—Y Network is employed in this research work as a dual encoder and single decoder architecture. One encoder is dedicated to extracting the feature from the fundus image, and another one is dedicated to extracting textural features from the local binary pattern. The model is trained on the IDRiD dataset. When comparing to other models trained in the IDRiD dataset, the proposed methods exhibit an accuracy of 99.87% for MA, 99% for HE, 99.42% for EX, and 99.85% for SE.

### 2.2 Attention network-based retinopathy segmentation

A cross-disease attention network model is introduced in Li X. et al. (2020) for grading both DR and Diabetic Macular Edema (DME) disease. Two different attention models are used for detecting DR and DME along with the loss function. They trained and extracted both spatial and channel features using the ISBI 2018 IDRiD dataset with the F1-score of 91.2% and 72.4% for DR and DME. A compound scaling-based encoder-decoder network is developed in Yi et al. (2024) for segmenting the diabetic retinopathy region. In the bottleneck layer, they applied depth-wise element multiplication, in addition, they extracted both the spatial and channel features for accurate segmentation. Deep Diabetic Retinopathy model is implemented in Mayya et al. (2021) for predicting the progression of DR based on time series.

An image-to-image translation network model is implemented in Shao et al. (2023) for fundus retinal image segmentation. In addition, the Gradient-Vector-Flow Loss function is deployed with cycleGAN for accurate retinal fundus segmentation. PLVS-Net was introduced in Arsalan et al. (2023) for segmenting the retinal vessel using prompt blocks. Prompt block helps to extract the required features using convolution kernels, depth-wise convolutions, and regular convolutions. In Gao et al. (2024), two sub-network models were implemented, namely, BiSeNet V2 and BiSeNet V2 Pro for detecting multiple lesion segmentation using a fundus image. Their proposal claims to be lightweight and addresses segmenting lesions of different origins and characteristics. An attention module was deployed at the semantic branch of V2 to enhance the refined features. BiSeNet V2 pro performance is measured using various metrics like sensitivity, Intersection over Union (IoU), and Dice coefficient, achieving the score of 12.17%, 11.44%, and 8.49%, respectively (Gautam and Shanker, 2025; Zhou et al., 2021).

CMNet model (Guo Y. et al., 2024) focused on the grading of diabetic retinopathy along with the diagnosis. In the CMNet model, they implemented a triple attention module, a cascading context fusion module, and a balance attention module. The purpose of these modules is to improve the selection and focus on the edge details. Their model is trained using the IDRiD dataset, which consists of 81 fundus images with a pixel size of 2848 X 4288. The quantitative performance of the CMNet model achieves the value of 0.6710, 0.4563, and 0.6211 for two lesion segmentations, which they claim that the percentage improvement of 16.2%, 4.74%, and 19.94% for mean Area Under Curve (mAUC), mean IOU, and mean Dice coefficient.

In Wang et al. (2023), CLC-Net is developed to segment lesions of fundus image; their proposal comprises a collaborative architecture, which comprises a contextual branch and a local branch. An attention mechanism is introduced in this system to fuse feature maps extracted from all decoders to combine informative features. In this research work, three datasets are used and compared with eight deep learning techniques, such as SegNet, Attention U-Net, DeepLab V3+, UNet++, DUpsample, CE-Net, CPF-Net, and L-Seg. When comparing with the results of different models using the iDRid dataset, the proposed method shows a better result for MA segmentation, with an accuracy of 0.546 and a mean of 0.677.

### 2.3 Transformer-based diabetic retinopathy segmentation

In recent days, transformers have played a major role in improving the accuracy of results over CNN models. In Nazih et al. (2023), the Vision Transformer (ViT) model is implemented for capturing long-range dependencies of retinal features using the Fine Grained Annotated DR dataset. They used a patch size of 32 while training the ViT model and achieved the F1-score value of 80.4%. A saliency-guided, self-supervised transformer is proposed in Huang et al. (2024), which consists of two learning tasks. 1. A saliency-guided model that helps to remove the patches from the encoder and helps to focus only on the salient regions. 2. A query-based encoder is deployed to predict saliency-segmented information for accurate grading.

A relation transformer block is proposed in Huang et al. (2022) for segmenting a multi-lesion region from the diabetic retinal images. In addition, a cross-attention transformer and a global transformer block are deployed to extract the lesion and vessel features from the given input images. The IDRiD dataset is used to train their model, and it achieves a precision score of 0.9024. In Oulhadj et al. (2024), they predict diabetic retinopathy; the author proposed a technique implemented with a vision transformer and a modified capsule network. The techniques involved in this algorithm contain power law transformation, contrast-limited adaptive histogram equalization, vision transformer, and capsule network. Finally, the result is programmed to classify the image as healthy, mild, moderate, or severe. APTOS, EyeSPACS, Messidor-2, and DDR datasets are used in this research work. The results of the model are compared with the state of the art, and it is identified that the accuracy obtained for the APTOS dataset is 88.18%.

Guo and Ji (2024), they developed an automated model for diagnosing the lesions and classifying the images pertaining to diabetic retinopathy. They introduced concepts referred to as random color transformation and single domain generalization for accurate segmentation. A dilated convolution net is implemented in Shang et al. (2024) with a three-layer architecture, and the segmentation is attained by extracting crucial features from all the levels of the feature map. The concepts referred to as positive sequence block and reverse sequence block are incorporated in each layer. A non-linear feature extraction module is introduced into the system to adjust the semantic information loss. Comparison is provided for modular ablation, deep ablation, and loss function experimentation. The accuracy achieved in the model is claimed to be 96.96%, 97.67%, 97.81%, and 97.40% for DRIVE, CHASEDB1, STARE, and HRF datasets.

The RMCA U-Net model is designed with a U-shaped framework with a residual structure in Fu et al. (2023). Multi-scale feature fusion is deployed with an improved channel attention mechanism to segment sparse small lesions. Multi-resolution contextual network (Khan T. M. et al., 2023) to extract the multi-scale features, particularly to perform vessel segmentation in retinal images. This model also incorporates the training in an adversarial setup, leading to an improvement in the foreground segmentation. SDDC-Net (Yang et al., 2023) performs retinal vessel segmentation from a fundus image. Their proposed system is claimed to have improved perpetual sensitivity for thin blood. DRIVE, STARE, and CHASEDB1 datasets are used to test and train the model. The author claims the improvement of sensitivity, accuracy, and F1 score to be 10.66 %, 1.73 %, and 1.47%, respectively, for the DRIVE dataset when comparing to the baseline U-Net.

ARSA-Unet (Xie et al., 2024) is introduced to replace the convolution layer with a feature screening fused module, which helps to improve the learning efficiency. DRIVE, STARE, and CHASE datasets are used to train their proposed model. Optimized wavelets are introduced in Venkaiahppalaswamy et al. (2023) to detect diabetic retinopathy. A cross-guided bilateral filter is used in pre-processing, and a wavelet-based chimp optimization algorithm is used for feature extraction. Binocular Siamese-based AlexNet, GoogleNet, and SVM are used for classification. The proposed work is claimed to have an accuracy of 94 % and 94.83 % in DB0 and DB1 datasets, respectively.

The visionDeep model is developed in Chandra Joshi et al. (2024) for both segmentation and classification of fundus images. Initially, they segmented the retinal vessel by implementing a bi-directional feature pyramid and a U-Net backbone. Secondly, they extracted the high-level and fine-grained characteristics using a six-layer depth network of an end-to-end encoder-decoder. The blood vessel segmentation result indicated by them is 97.73 % of accuracy and 89.90 % of dice coefficient. A graph convolution network is employed in Wang et al. (2024) to precisely segment the vessel in a complex situation. U-Net is employed in their system to enhance the segmentation. The partial class activation mapping will enable the transmission between nodes and activate vessel features. The hierarchical diffusion model is used for segmenting the blood vessels in the fundus image (Huang and Liu, 2024). It has a combination of a dual guidance module and a refinement guidance module. The purpose of the dual guidance module is to create an outline of the vascular system in the image. The refinement guidance module helps to segment more accurately.

Based on the above made survey, various existing U-Net variants, attention-based networks, and transformer-based models have shown promising performance in diabetic retinopathy analysis. However, most of these methods focus on improving only one aspect of feature learning, such as local feature extraction, attention mechanisms, or global context modeling. To overcome these limitations, the proposed ARTNet combines the Adaptive Channel-wise Feature Network (ACFNet), Ophthalmic Region-Aware Attention Network (ORAANet), and Retinal Patch Aggregation Encoder Network (RPAENet) into a unified framework. By integrating these complementary components, ARTNet effectively captures both fine-grained retinal lesions and global contextual information, resulting in more accurate lesion localization and diabetic retinopathy classification.

## 3 Proposed Adaptive channel-wise and Region-aware Transformer Network (ARTNet)

**Figure 1:**
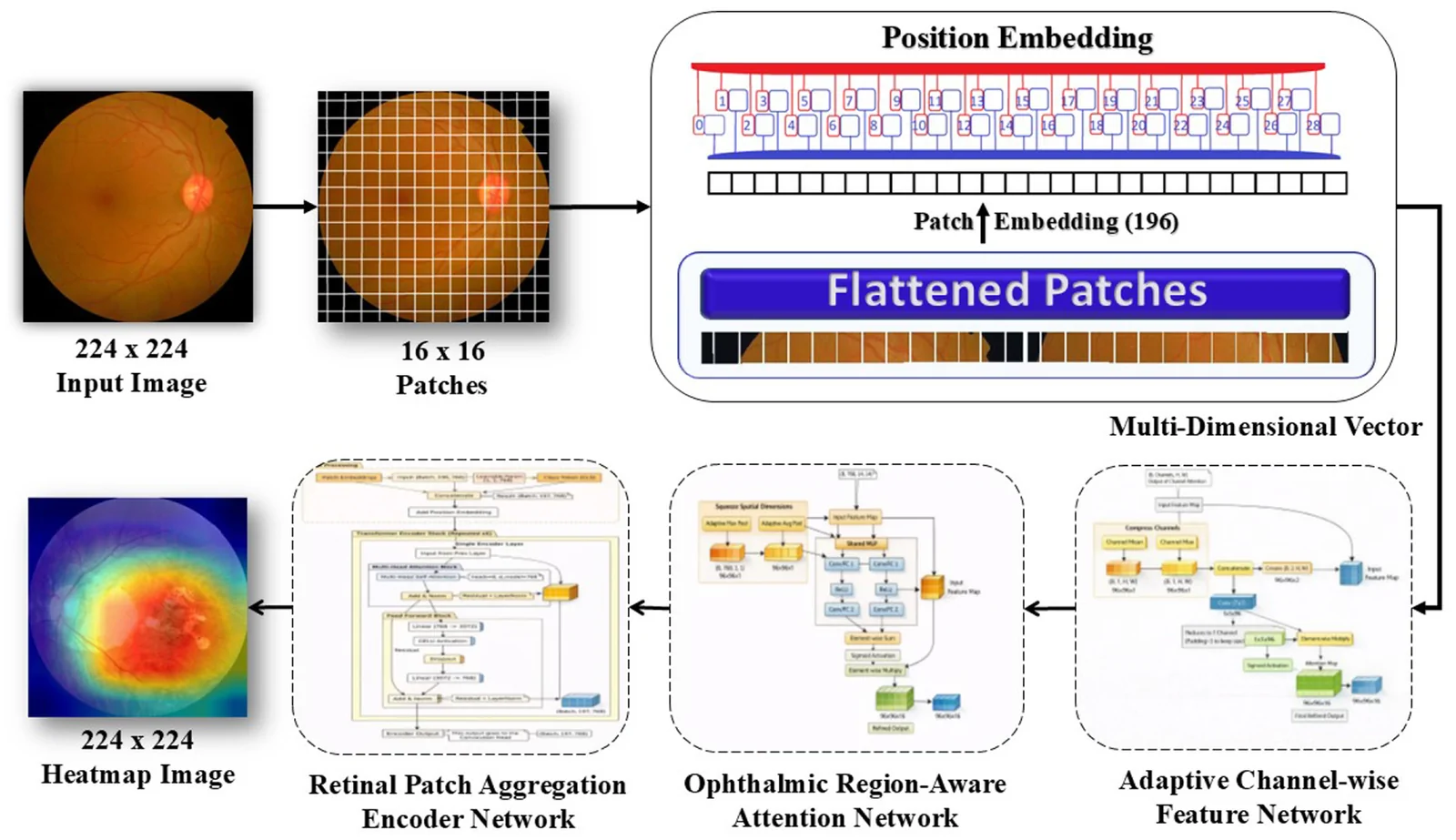
Illustrates the overview architecture of the proposed ARTNet model. A 224 × 224 fundus image is divided into 16 × 16 patches and embedded as 196 tokens with positional encoding. The features are sequentially refined by the Adaptive Channel-wise Network, Ophthalmic Region-Aware Attention, and Retinal Patch Aggregation Encoder network, producing a heatmap highlighting DR-affected salient regions.

The proposed ARTNet comprises three interconnected sub-networks for detecting and segmenting diabetic retinopathy lesions in retinal fundus images. It integrates three key components—the Adaptive Channel-wise Feature Network, the Ophthalmic Region-Aware Attention Network, and the Retinal Patch Aggregation Encoder Network—to capture both local and global retinal features, enabling accurate and robust lesion segmentation. The retinal fundus image of size 224 × 224 pixels is provided as input. The image is divided into non-overlapping patches of size 16 × 16, resulting in a total of 196 patches. Each patch is projected into a high-dimensional embedding and combined with positional encoding to preserve spatial context. This process forms a multi-dimensional vector representation. Then, the embeddings are passed through the Adaptive Channel-wise Feature Network (ACFNet). ACFNet incorporates an adaptive channel reweighting mechanism. This mechanism emphasizes diagnostically relevant retinal features, such as microaneurysms, hemorrhages, and exudates. It simultaneously suppresses irrelevant background information. The refined embeddings are processed by the Ophthalmic Region-Aware Attention Network (ORAANet), which highlights critical retinal regions, including lesions and abnormal vasculature, to guide the network's focus. Finally, the extracted features are aggregated by a Retinal Patch Aggregation Encoder Network (RPAENet) based on multi-head self-attention. RPAENet captures long-range dependencies and global context across the retina, enhancing the model's ability to segment both subtle and widespread abnormalities. Convolutional operations are applied afterward to preserve local spatial hierarchies and refine feature maps. Figure 1 illustrates the overall architecture of the proposed ARTNet, showing the sequential integration of the Adaptive Channel-wise Feature Framework, Ophthalmic Region-Aware Attention Framework, and Retinal Patch Aggregation Encoder for accurate segmentation of the diabetic retinopathy region. Table 1 presents the symbol notations along with their corresponding descriptions.

**Table 1:** Notation and description for the proposed ARTNet methodology.

Notation	Description
*I*	Input retinal fundus image of size 224 × 224 × 3
*P*_*i*_	*i*-th non-overlapping image patch of size 16 × 16 × 3
*d*_raw_	Flattened patch dimension *C*·*p*^2^
*d*	Projected patch embedding dimension (768)
**z**_*i*_	Embedding vector of patch *P*_*i*_
${\text{e}}_{i}^{\text{pos}}$	Positional encoding for patch *i*
**Z**	Grid of patch embeddings before attention
**Z**′	Output of ACFNet (channel-refined embeddings)
**Z**″	Output of ORAANet (region-refined embeddings)
**Z**_flat_	Flattened patch embeddings input to RPAENet
**h**_[CLS]_	[CLS] token embedding capturing global retinal context
**h**_grid_	Structured feature grid after 3 × 3 convolution
*g*_GAP_	Vector after Global Average Pooling (GAP)
*f*_cls_	Fully connected classification head
ŷ	Predicted class probability vector for 5 DR stages
*y*	Ground truth class label
ĉ	Predicted class label ∈{0, 1, 2, 3, 4}
**θ**	ARTNet trainable parameters
$L_{\text{focal}}$	Class-balanced focal loss
$L_{\text{CE}}$	Cross-entropy loss
*p*_*t*_	Predicted probability for the true class
$A_{\text{train}},A_{\text{val}}$	Accuracy on training and validation sets
$L_{\text{val}},L_{\text{avg}}$	Validation and average training loss
*L*_train_, *L*_val_, *L*_test_	DataLoaders for training, validation, and testing sets

### 3.1 Pre-processing

**Figure 2:**
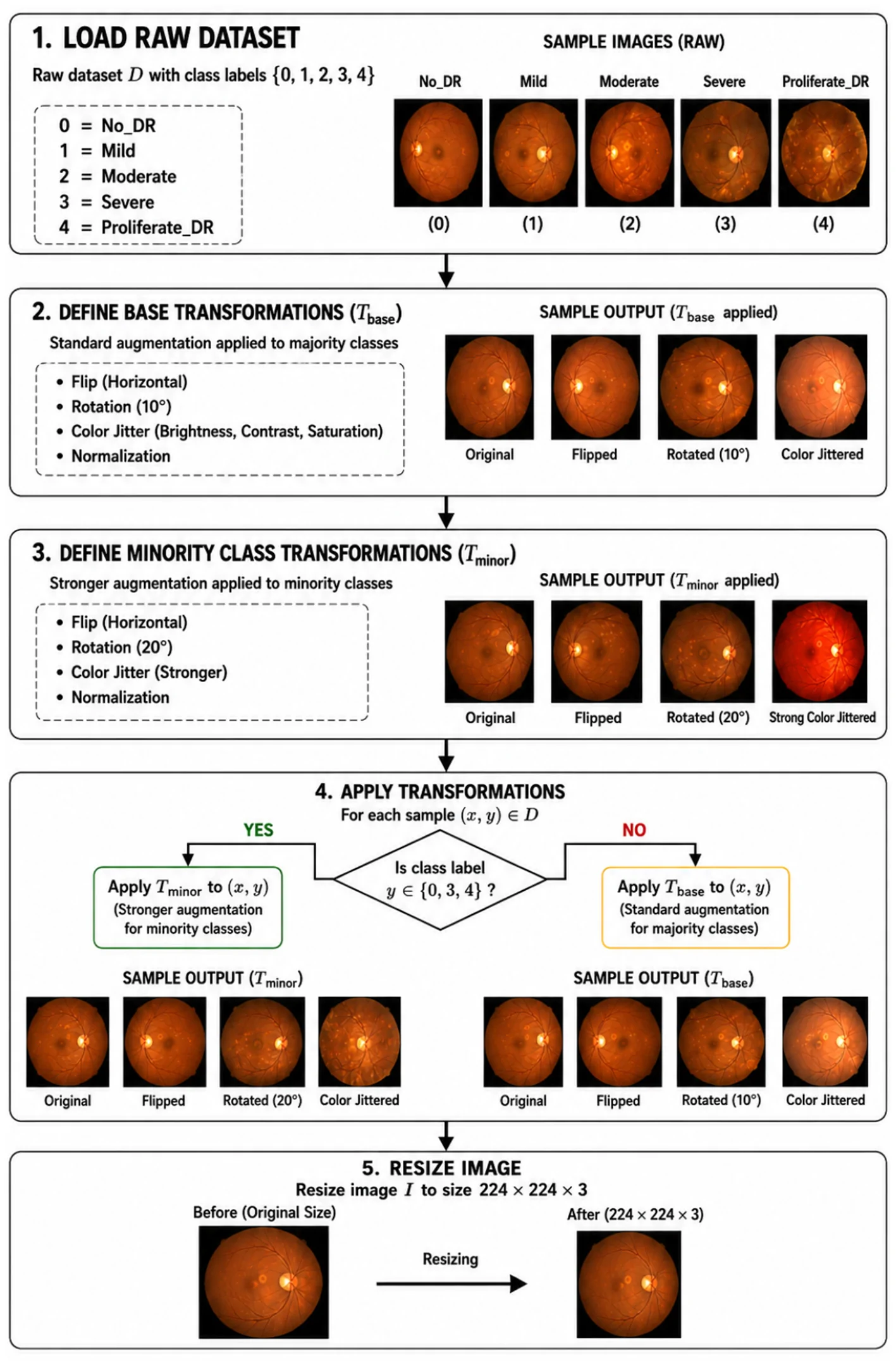
Illustrates the data pre-processing pipeline showing dataset loading, majority/minority class-specific augmentation, and conditional transformation selection. All processed retinal images are resized to 224 × 224 × 3.

The data pre-processing pipeline loads the raw dataset *D*, consisting of retinal images with class labels from 0 to 4, corresponding to different stages of diabetic retinopathy. Two image transformation strategies are defined: base transformations (*T*_base_) and minority class transformations (*T*_minor_). The base transformations include flipping, small rotations of 10°, color jittering, and normalization. For minority classes, stronger augmentations are applied. These include flipping, larger rotations of 20°, color jittering, and normalization to increase data diversity. Each image is transformed based on its class label: images from minority classes (0, 3, and 4) use *T*_minor_, whereas the remaining classes use *T*_base_. All images are resized to a uniform resolution of 224 × 224 × 3. The dataset is split into training, validation, and testing sets using an 80:10:10 ratio. To address class imbalance, class weights (*W*_*c*_) are computed using a balanced strategy, and per-sample weights are assigned accordingly. To mitigate class imbalance, stronger augmentation was applied to minority classes, while balanced class weights and a WeightedRandomSampler ensured uniform sample representation during training. Furthermore, a class-balanced focal loss improved minority-class learning by reducing bias toward majority classes and enhancing overall model generalization. Algorithm 1 outlines the pre-processing procedure performed. Figure 2 provides a schematic representation of the data pre-processing pipeline, including class-specific augmentation and image standardization prior to model training.

Algorithm 1 **Data pre-processing.**

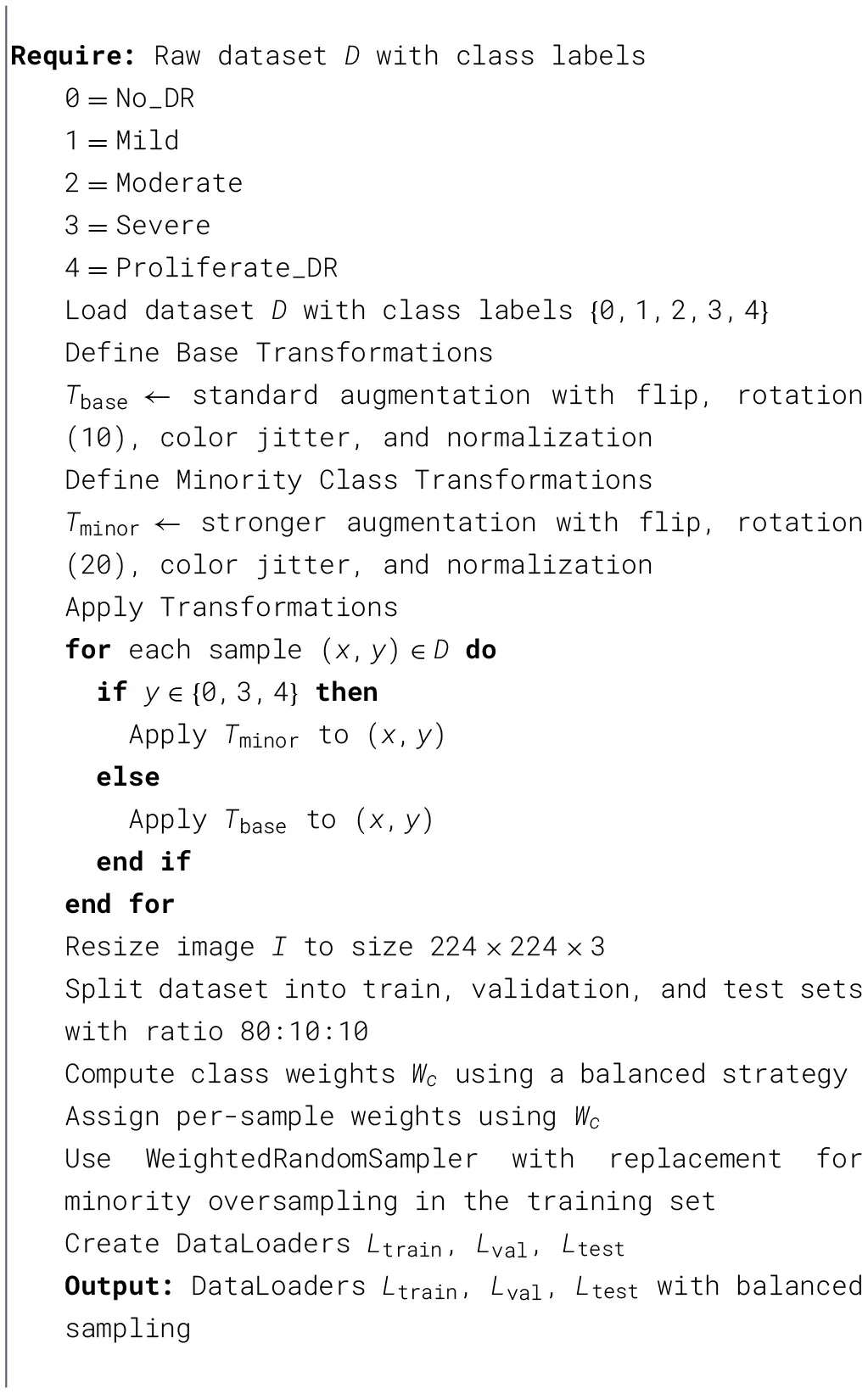

### 3.2 Feature embedding

The input to the model is a high-resolution fundus image *I*∈ℝ^224 × 224 × 3^. The size 224 × 224 represents the image dimensions, and 3 corresponds to the RGB color channels. To process the image efficiently, it is divided into smaller, non-overlapping square patches ${\left\{P_{i}\right\}}_{i=1}^{P}$. Each patch $P_{i}\in {\mathbb{R}}^{16\times 16\times 3}$ is a small cropped part of the original image. These patches capture localized retinal features such as blood vessels, microaneurysms, or lesions. Each patch *P*_*i*_ is transformed into a vector $z_{i}\in {\mathbb{R}}^{d}$ by a learnable linear projection layer *P*_proj_. This layer maps raw pixel data into a higher-dimensional feature space with *d* = 768. It extracts meaningful patterns from each patch independently. Patches alone do not carry positional information. To fix this, positional encodings $e_{i}^{\text{pos}}$ are added to each patch embedding. These encodings enable the model to capture the spatial arrangement of image patches. Extraction of spatial features helps identify lesion locations that influence diagnostic interpretation. After embedding and positional encoding, the patches are passed to the Adaptive Channel-wise Feature Network. Algorithm 2 highlights the sequential steps involved in training the proposed ARTNet model for DR segmentation.

Algorithm 2 **Training procedure of ARTNet.**

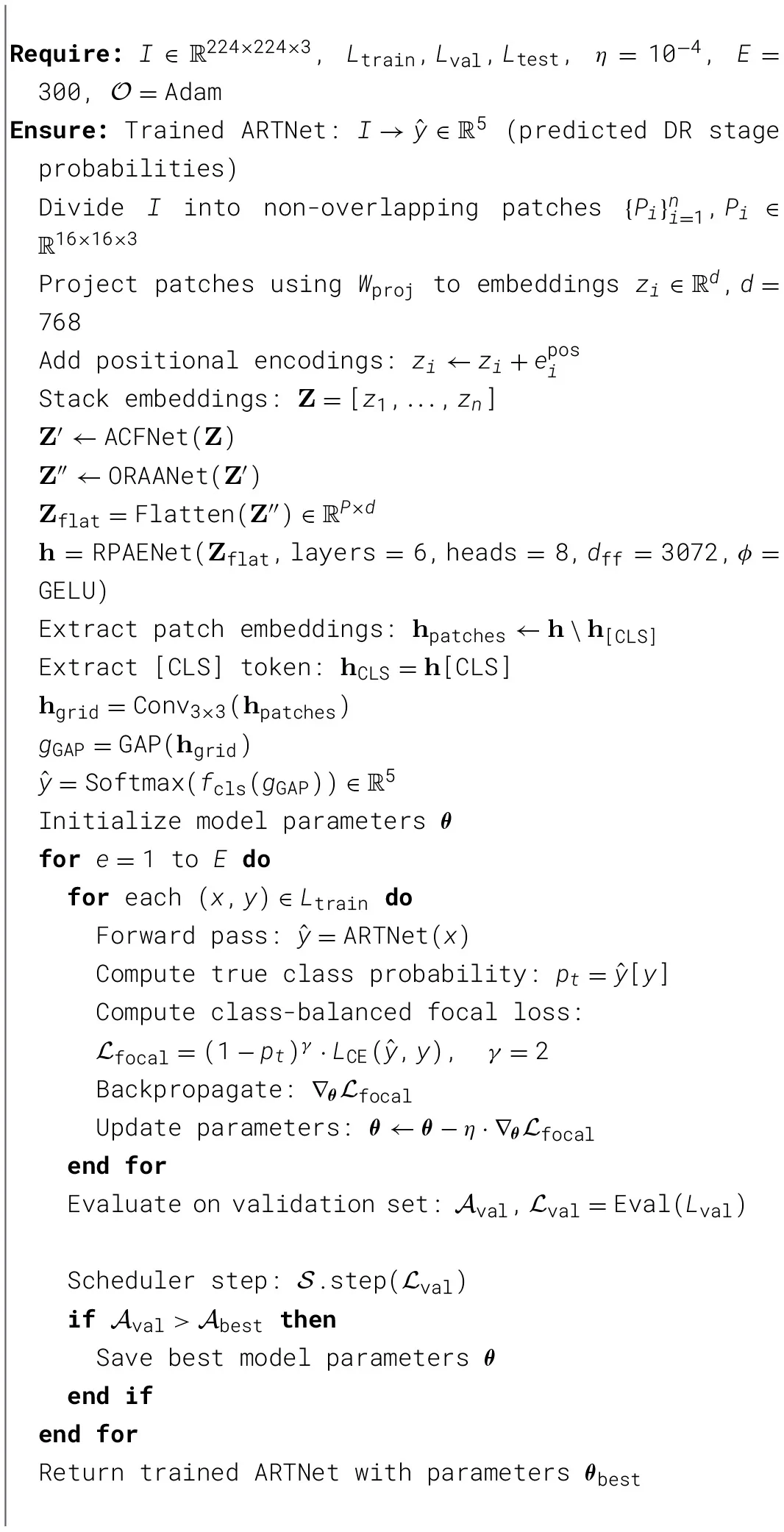

### 3.3 Adaptive Channel-wise Feature Network (ACFNet)

The ACFNet functions on an intermediate feature tensor **F**∈ℝ^*B*×*C*×*H*×*W*^, where *B* denotes the batch size, *C* represents the number of channels, and *H*×*W* indicates the spatial resolution. To capture global contextual information, the spatial dimensions are compressed through parallel adaptive average pooling and adaptive max pooling operations. This spatial squeezing generates channel-wise descriptors ${\text{f}}_{avg},{\text{f}}_{max}\in {\mathbb{R}}^{B\times C\times 1\times 1}$, summarizing the global distribution of responses across each channel. The descriptors are then processed by a shared multi-layer perceptron (MLP), implemented using 1 × 1 convolutions (or fully connected layers), producing transformed representations using Equation 1:


$$\begin{array}{l} {\text{z}}_{avg}=W_{2}(\delta \left(W_{1}\left({\text{f}}_{avg}\right)\right)),\;{\text{z}}_{max}=W_{2}(\delta \left(W_{1}\left({\text{f}}_{max}\right)\right)) \end{array}$$ (1)

where *W*_1_ and *W*_2_ are learnable parameters and δ(·) denotes the ReLU activation. This transformation models the non-linear inter-channel dependencies and enhances discriminative feature interactions. The outputs of both branches are fused through element-wise summation to form a unified descriptor using Equation 2


$$\begin{array}{l} \text{z}={\text{z}}_{avg}+{\text{z}}_{max} \end{array}$$ (2)

which is subsequently normalized using a sigmoid function to generate channel attention coefficients as indicated in Equation 3


$$\begin{array}{l} {\text{M}}_{c}=\sigma \left(\text{z}\right),\;{\text{M}}_{c}\in {\mathbb{R}}^{B\times C\times 1\times 1} \end{array}$$ (3)

These coefficients are applied to the original feature tensor via element-wise multiplication, yielding recalibrated features using Equation 4


$$\begin{array}{l} {\text{F}}^{\prime }={\text{M}}_{c}⊙\text{F} \end{array}$$ (4)

**Figure 3:**
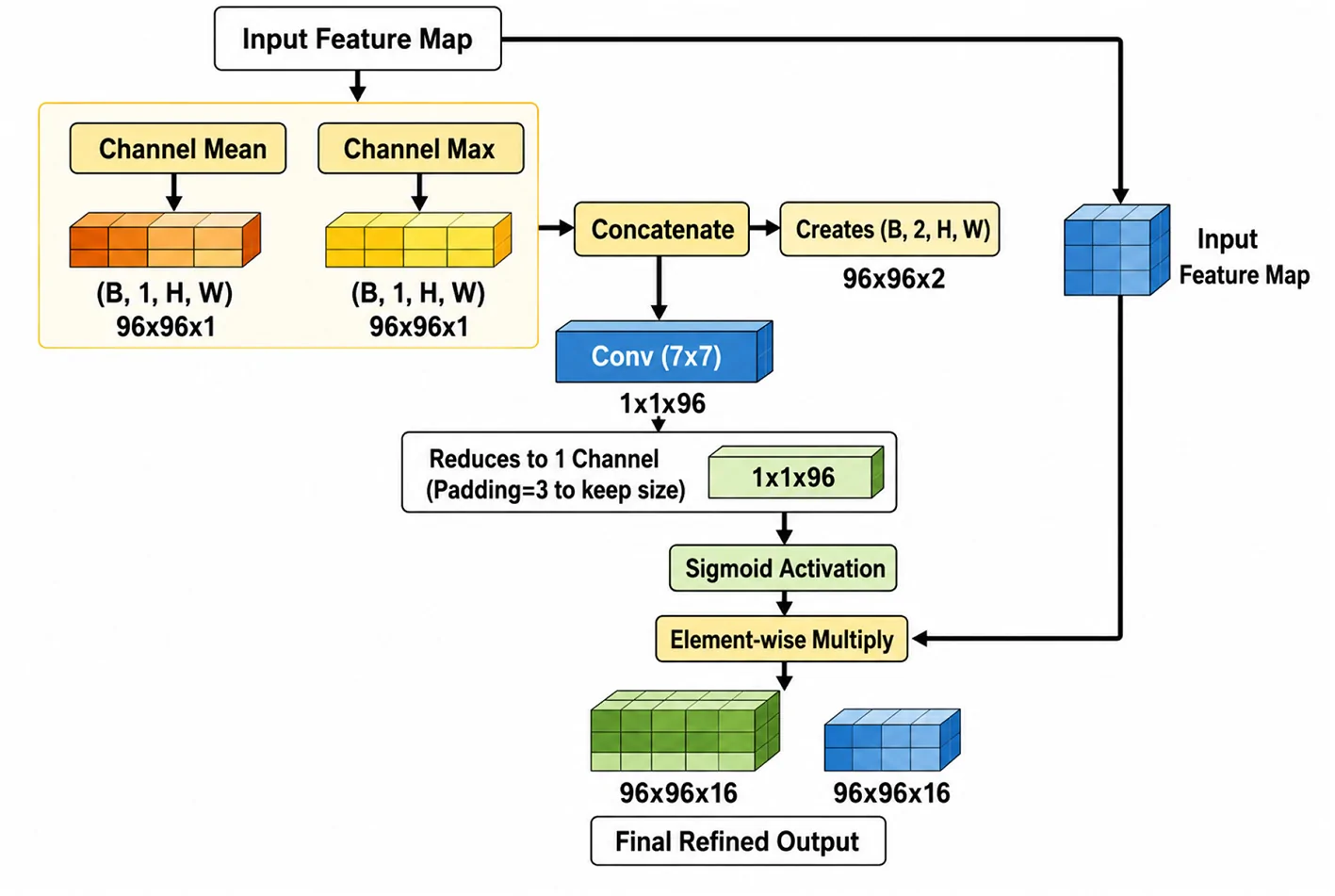
Adaptive Channel-wise Feature Network employing mean and max pooling, 7 × 7 convolution, sigmoid activation, and feature recalibration.

This feature recalibration emphasizes informative channels while suppressing less relevant responses, thereby improving representational quality for subsequent processing stages. Figure 3 illustrates the Adaptive Channel-wise Feature Network (ACFNet).

### 3.4 Ophthalmic Region-Aware Attention Network (ORAANet)

**Figure 4:**
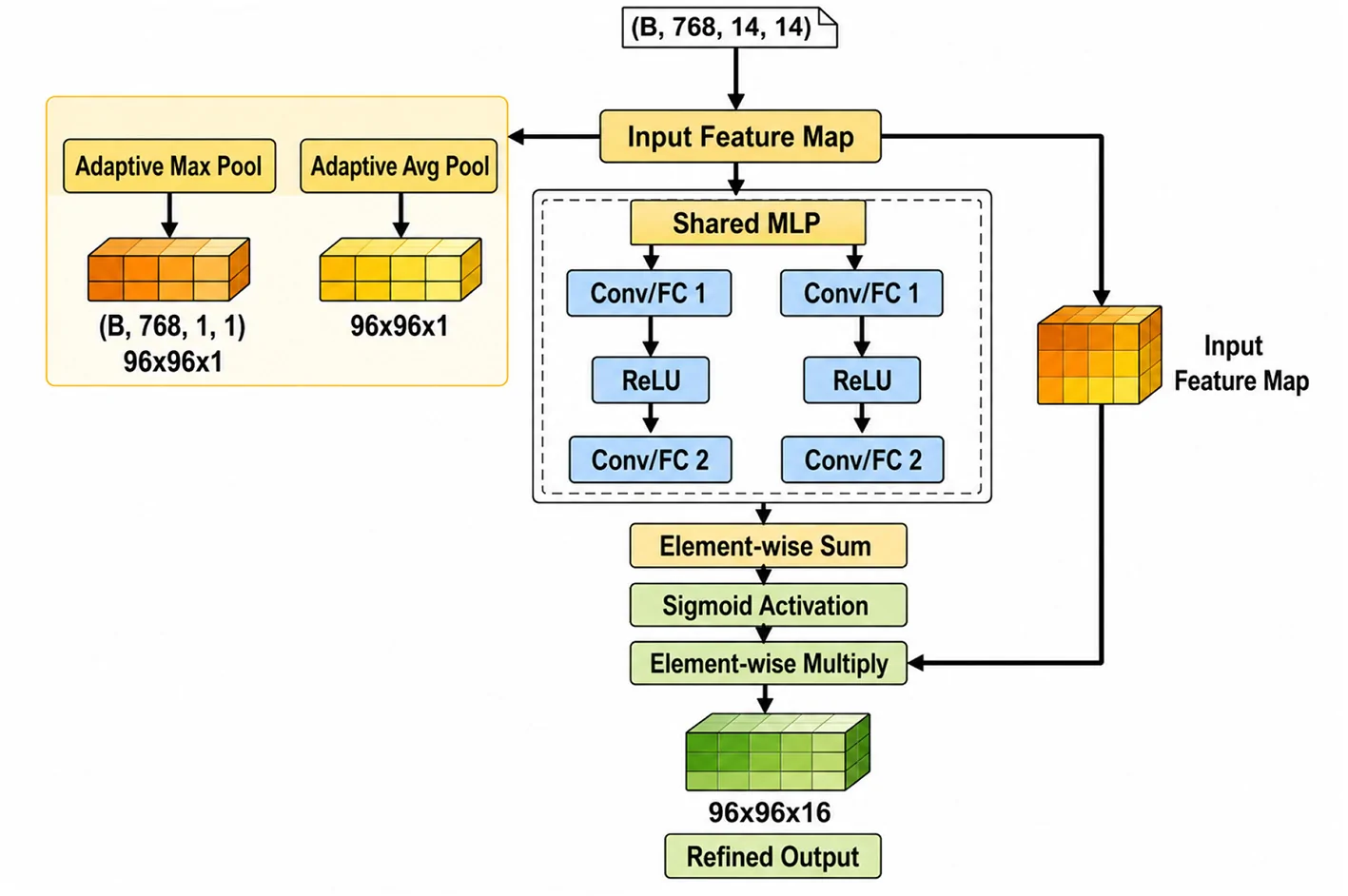
Ophthalmic Region-Aware Attention Network: compresses spatial dimensions using adaptive pooling and applies a shared MLP with ReLU and sigmoid gating to extract features from DR-affected regions.

ORAANet takes as input the refined feature map generated by ACFNet, **F**′∈ℝ^*B*×*C*×*H*×*W*^. This feature map is processed by the Region-Aware Attention module. This sub-network identifies diagnostically relevant retinal regions, as illustrated in Figure 4. ACFNet extracts discriminative feature representations, whereas the region-aware attention module localizes clinically meaningful spatial patterns. In ACFNet, region descriptors are obtained by aggregating channel information using parallel channel-wise average and max pooling operations. This helps to produce two ophthalmic region-aware representations ${\text{s}}_{avg},{\text{s}}_{max}\in {\mathbb{R}}^{B\times 1\times H\times W}$, capturing complementary contextual cues regarding distributed responses and prominent activations across retinal regions. These region descriptors are concatenated along the channel dimension to form a unified ophthalmic region-aware feature representation as indicated in Equation 5.


$$\begin{array}{l} \text{S}=\text{Concat}\left({\text{s}}_{avg},{\text{s}}_{max}\right)\in {\mathbb{R}}^{B\times 2\times H\times W} \end{array}$$ (5)

The aggregated representation is further processed by a 7 × 7 convolution to capture contextual regional dependencies and generate a region-aware attention map as computed using Equation 6.


$$\begin{array}{l} {\text{M}}_{r}=\sigma \left(f^{7\times 7}\left(\text{S}\right)\right) \end{array}$$ (6)

where *f*^7 × 7^(·) denotes convolution with padding to preserve ophthalmic region-aware resolution and σ(·) represents sigmoid activation. The resulting ophthalmic region-aware attention map ${\text{M}}_{r}\in {\mathbb{R}}^{B\times 1\times H\times W}$ highlights pathological retinal areas such as lesion clusters, abnormal vasculature, and structural irregularities. The resulting attention map is applied to the incoming feature map through element-wise multiplication, as indicated in Equation 7.


$$\begin{array}{l} {\text{F}}^{\prime \prime }={\text{M}}_{r}⊙{\text{F}}^{\prime } \end{array}$$ (7)

This helps to generate region-refined features that emphasize clinically significant retinal areas while suppressing background noise. It improves localization performance and achieves a higher accuracy rate.

### 3.5 Retinal Patch Aggregation Encoder Network and hierarchical feature learning

The region-refined feature representations obtained from ORAANet is indicated in Equation 8.


$$\begin{array}{l} {\text{F}}^{\prime \prime }\in {\mathbb{R}}^{B\times C\times H\times W} \end{array}$$ (8)

where *B*, *C*, *H*, and *W* denote the batch size, number of channels, height, and width, respectively. These features are provided as input to the Retinal Patch Aggregation Encoder Network (RPAENet); the region-refined features dimensions of **F**″ in Equation 8 are partitioned into *P* non-overlapping patches and reshaped into a sequence of embeddings as represented in Equation 9.


$$\begin{array}{l} \text{Z}=\text{Reshape}\left({\text{F}}^{\prime \prime }\right)\in {\mathbb{R}}^{B\times P\times d} \end{array}$$ (9)

where the total number of patches is defined in Equation 10.


$$\begin{array}{l} P=\frac{H\times W}{p^{2}}, \end{array}$$ (10)

where *p* denotes the patch size. The embedding dimension is given in Equation 11 to the flattened region-aware channel representation.


$$\begin{array}{l} d=C\cdot p^{2} \end{array}$$ (11)

Each flattened patch $x_{i}\in {\mathbb{R}}^{d_{\text{raw}}}$ from ORAANet is linearly projected into a higher-dimensional embedding using a learnable matrix $W_{p}\in {\mathbb{R}}^{d_{\text{raw}}\times d}$ and it is computed using Equation 12.


$$\begin{array}{l} z_{i}^{\prime }=x_{i}W_{p} \end{array}$$ (12)

where *d* is the projected embedding dimension. The resulting patch embeddings ${\left\{z_{i}^{\prime }\right\}}_{i=1}^{P}$ are then arranged sequentially to form the flattened patch sequence, and it is computed using Equation 13.


$$\begin{array}{l} Z=\left[z_{1}^{\prime },z_{2}^{\prime },\ldots ,z_{P}^{\prime }\right]\in {\mathbb{R}}^{P\times d} \end{array}$$ (13)

The flattened patch sequence *Z* serves as input to the Retinal Patch Aggregation Encoder Network (RPAENet). It captures long-range dependencies between spatially distant regions of the retina. Such dependencies are crucial for diabetic retinopathy analysis, since small lesions in one area may relate to patterns in other regions. The RPAENet is composed of 6 layers. Each layer contains 8 attention heads, a hidden dimension of *d*_*f*_ = 3072, and uses the GELU activation function. For each attention head, self-attention is computed using Equation 14.


$$\begin{array}{l} \text{Attention}\left(Q,K,V\right)=\text{Softmax}\left(\frac{QK^{T}}{\sqrt{d_{k}}}\right)V \end{array}$$ (14)

where *Q, K, V* are the query, key, and value matrices derived from *Z*, and *d*_*k*_ is the dimension of each head. After processing through the encoder, the output feature sequence is obtained as indicated in Equation 15.


$$\begin{array}{l} h\in {\mathbb{R}}^{R\times d} \end{array}$$ (15)

where *R* is the number of patches [including the [CLS] token]. The encoder produces contextualized feature embeddings for all input patches as [CLS] token as indicated in Equation 16.


$$\begin{array}{l} h_{\text{CLS}}=h\left[\text{CLS}\right] \end{array}$$ (16)

>The [CLS] token aggregates global retinal context and provides discriminative semantic information for diabetic retinopathy grading. To complement this global representation with local spatial structure, 3 × 3 convolutional operations (Conv_3 × 3_) are applied to the transformer outputs as indicated in Equation 17.


$$\begin{array}{l} h_{\text{grid}}={\text{Conv}}_{3\times 3}\left(h\right) \end{array}$$ (17)

**Figure 5:**
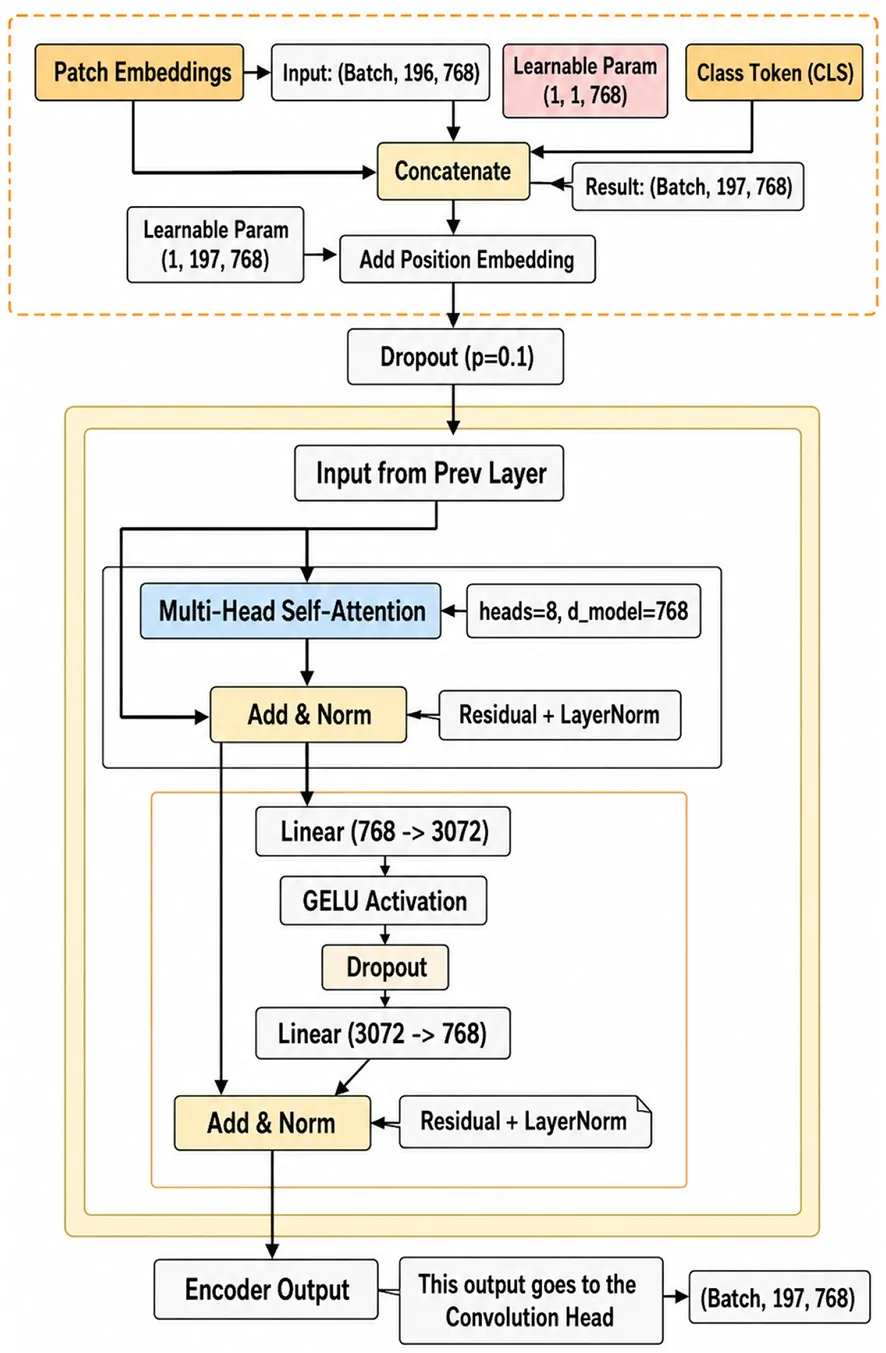
Retinal Patch Aggregation Encoder Network with embedded patches and class token processed through six transformer layers featuring multi-head self-attention and feed-forward blocks.

This operation generates a structured feature grid. It preserves spatial relationships and enhances local detail representation. The integration of transformer-driven global reasoning with convolutional local refinement facilitates the detection of fine lesions, such as microaneurysms. It also captures larger abnormalities, such as hemorrhages. This improves segmentation and grading performance. Figure 5 represents the network architecture of the Retinal Patch Aggregation Encoder Network.

### 3.6 Classification and optimization strategy

After hierarchical feature extraction using the RPAENet and convolutional refinement, the resulting feature grid *h*_grid_ is reduced using Global Average Pooling (GAP) to obtain the feature vector, as indicated in Equation 18.


$$\begin{array}{l} g_{\text{GAP}}^{\left(k\right)}=\frac{1}{H\times W}\overset{H}{\underset{i=1}{\sum }}\overset{W}{\underset{j=1}{\sum }}h_{\text{grid}}^{\left(k\right)}\left(i,j\right),\;k=1,\ldots ,d, \end{array}$$ (18)

where *H* and *W* denote the spatial height and width of the ophthalmic region-aware feature grid. Global Average Pooling aggregates spatial (region-aware) information within each adaptive channel-wise feature map while preserving salient semantic responses. This helps to reduce the feature dimensionality and computational complexity.

The feature vector *g*_GAP_ is passed through a fully connected classification head *f*_cls_. Then it is passed to the Softmax function to predict class probabilities for the five diabetic retinopathy stages, as represented in Equation 19.


$$\hat{y}=\text{Softmax}(f_{\text{cls}}(g_{\text{GAP}})))\in {\mathbb{R}}^{5}$$ (19)

where ŷ_*i*_ denotes the predicted probability of the *i*_*th*_ stage. Softmax function computed using Equation 20.


$$\begin{array}{l} {ŷ}_{i}=\frac{\exp\left(z_{i}\right)}{\sum_{j=1}^{5}\exp\left(z_{j}\right)} \end{array}$$ (20)

with *z*_*i*_ indicates the raw logit for class *i*. To handle class imbalance, a local contrastive cross-entropy loss is used, and it is indicated in Equation 21.


$$\begin{array}{l} L_{\text{local}}={\left(1-p_{t}\right)}^{\gamma }\cdot L_{\text{CE}}\left(ŷ,y\right)\;\gamma =2 \end{array}$$ (21)

where *p*_*t*_ is the predicted probability of the ground-truth class *y*, *L*_CE_ is the standard cross-entropy loss, and γ is a focusing parameter that reduces the contribution of easy examples while emphasizing minority-class samples.

### 3.7 Loss function

To address an inherently imbalanced dataset, ARTNet is optimized using a class-balanced focal loss. For each training sample (*x, y*), the network outputs a class probability vector ŷ = ARTNet(*x*), and the probability of the true class is denoted as *p*_*t*_ = softmax(ŷ)[*y*]. The focal loss is derived using Equation 22.


$$\begin{array}{l} L_{\text{focal}}={\left(1-p_{t}\right)}^{\gamma }\cdot L_{\text{CE}}\left(ŷ,y\right) \end{array}$$ (22)

where $L_{\text{CE}}$ represents the standard cross-entropy loss. The model parameters are updated through gradient-based optimization as indicated in Equation 23.


$$\begin{array}{l} \theta \leftarrow \theta -\eta \cdot \nabla_{\theta }L_{\text{focal}} \end{array}$$ (23)

where η denotes the learning rate and θ represents trainable parameters.

## 4 Experiments

This section presents the experimental setup used to evaluate the proposed ARTNet model. The datasets employed for training and validation are first described. The evaluation metrics used to measure performance are then outlined. Finally, the implementation details are discussed.

### 4.1 Dataset

The proposed ARTNet was trained using only the Diabetic Retinopathy Detection dataset, with an 80% training and 20% testing split. From the training set, 10% was reserved for validation during model selection and hyperparameter tuning. The APTOS-2019 and IDRiD datasets were used only for external testing, with no fine-tuning or parameter updates, to evaluate the cross-dataset generalization capability of the proposed ARTNet model.

#### 4.1.1 Diabetic retinopathy detection dataset

We evaluated the performance of our proposed model using the Diabetic Retinopathy Detection dataset (Dugas et al., 2015), which contains high-resolution retinal fundus images suitable for deep learning applications. This dataset consists of 35,126 color retinal images, each labeled with one of five diabetic retinopathy severity levels: Class 0 (No DR), Class 1 (Mild), Class 2 (Moderate), Class 3 (Severe), and Class 4 (Proliferative DR). The dataset exhibits class imbalance, with Class 0 containing 25,810 images (73.5%), Class 1 containing 2,709 images (7.7%), Class 2 containing 5,131 images (14.6%), Class 3 containing 711 images (2.0%), and Class 4 containing 765 images (2.2%). Images are preorganized into class-specific directories for streamlined processing and resized as needed for model compatibility. The standard dataset split allocates 28,101 images (80%) for training, 7,025 images (20%) for testing, ensuring robust model evaluation across all severity levels. The Diabetic Retinopathy Detection dataset provides only image-level DR labels. Therefore, ARTNet performs DR classification, and its segmentation branch produces attention-guided lesion localization maps for qualitative visualization only. The dataset exhibits a highly imbalanced class distribution, with the majority of samples belonging to the No DR category. To mitigate the resulting training bias, class-aware augmentation, weighted sampling, balanced class weights, and class-balanced focal loss were jointly employed during model training.

#### 4.1.2 Other dataset

- APTOS-2019 Blindness Detection dataset (Rath, 2019) consists of 3,662 retinal images used to predict the severity of diabetic retinopathy. Each image is labeled with a severity level ranging from 0 (no diabetic retinopathy) to 4 (severe diabetic retinopathy). Our proposed ARTNet model is tested with 732 images. APTOS-2019 provides image-level severity labels only and was therefore used exclusively for classification evaluation.
- Indian diabetic retinopathy image (IDRiD) dataset (Herrera and Contributors, 2020) comprised of 516 high-resolution retinal fundus images with corresponding annotations for diabetic retinopathy (DR) grading and diabetic macular edema (DME). For model evaluation, the images are typically resized to 224 × 224 pixels, and the dataset includes both lesion-level and image-level labels covering five DR severity levels: No DR, Mild, Moderate, Severe, and Proliferative DR. The IDRiD dataset provides both image-level DR grades and pixel-level lesion annotations. The lesion annotations were used as the ground-truth masks for evaluating the segmentation performance of the proposed ARTNet using Dice, IoU, and MCC.

### 4.2 Evaluation metrics

The performance of the proposed model is evaluated using standard classification and segmentation metrics derived from the confusion matrix. Classification performance was evaluated using Accuracy, Precision, Recall, Specificity, F1-score, AUC, Macro-F1, and Weighted-F1. Segmentation performance was evaluated only on datasets providing pixel-level lesion annotations (IDRiD), using Dice coefficient, Intersection over Union (IoU), and Matthews Correlation Coefficient (MCC). The confusion matrix categorizes predictions into four groups:

- True Positive (TP) – Number of actual Diabetic Retinopathy affected regions that are predicted as affected regions.
- True Negative (TN) – Number of actual regions unaffected by Diabetic Retinopathy that are correctly predicted as unaffected regions.
- False Positive (FP) – Number of actual healthy regions incorrectly predicted as affected by Diabetic Retinopathy.
- False Negative (FN) – Number of actual Diabetic Retinopathy-affected regions that are not detected by the predicted segmentation.

Precision, Recall, F1-Measure, and Accuracy are computed using Equations 24–27.

- *Precision (* *P*_*ARTNet*_ *)*

$$\begin{array}{l} P_{ARTNet}=\frac{TP}{TP+FP} \end{array}$$ (24)

- *Recall (* *R*_*ARTNet*_ *)*

$$\begin{array}{l} R_{ARTNet}=\frac{TP}{TP+FN} \end{array}$$ (25)

- *F1 Score (F*1_*ARTNet*_*)*

$$\begin{array}{l} F1_{ARTNet}=2\cdot \frac{P\cdot R}{P+R} \end{array}$$ (26)

- *Accuracy (* *Acc*_*ARTNet*_ *)*

$$\begin{array}{l} Acc_{ARTNet}=\frac{TP+TN}{TP+TN+FP+FN} \end{array}$$ (27)

To qualitatively evaluate the proposed ARTNet model on diabetic retinopathy-segmented regions, various evaluation metrics are used. Intersection over Union (IoU) measures the degree of overlap between the predicted segmented diabetic retinopathy regions and the ground truth. It is derived using Equation 28. Dice coefficient measures the similarity between the predicted segmentation and the ground truth regions, computed using Equation 29, and Matthews Correlation Coefficient (MCC) evaluates the overall segmentation performance by accounting for both correct and incorrect predictions, and is computed using Equation 30.

- *Intersection over Union*_*ARTNet*_ *(IoU*_*ARTNet*_)

$$\begin{array}{l} \begin{array}{c} IOU_{ARTNet}=\left(\frac{TP}{TP+FP+FN}+\frac{TN}{TN+FP+FN}\right) \end{array} \end{array}$$ (28)

- *Dice Coefficient (* *D* *)*

$$\begin{array}{l} D_{ARTNet}=\frac{2TP}{2TP+FP+FN} \end{array}$$ (29)

- *Matthews Correlation Coefficient (* *MCC*_*ARTNet*_ *)*

$$\begin{array}{l} MCC_{ARTNet}=\frac{\left(TP\cdot TN\right)-\left(FP\cdot FN\right)}{\sqrt{\left(TP+FP\right)\left(TP+FN\right)}}\\ \;\times \frac{1}{\sqrt{\left(TN+FP\right)\left(TN+FN\right)}} \end{array}$$ (30)

### 4.3 Implementation details

The proposed ARTNet model is implemented using PyTorch (PyTorch Foundation, San Francisco, USA) with CUDA acceleration. The computational environment consisted of an Intel^®^ Xeon^®^ CPU operating at 2.20 GHz with four physical cores, 30 GB of system RAM, and an NVIDIA Tesla P100 GPU (NVIDIA Corporation, Santa Clara, USA) equipped with 16 GB HBM2 memory. The input images were resized to a fixed resolution of 224 × 224 × 3 before being fed into the network. The dataset was divided into 80% for training and 20% for testing, with a portion of the training set further allocated for validation to monitor generalization performance. The model is trained for 300 epochs with the Adam optimizer and a learning rate of 0.0001. The total training time is approximately 10 h and 15 min. During evaluation, batch-wise inference is performed on the test set, resulting in a total testing time of approximately 9.2 s. An average inference latency of 25.10 milliseconds per image. Table 2 shows the computational complexity of the proposed ARTNet model.

**Table 2:** Computational complexity and execution environment of the proposed ARTNet model.

Parameter	Technical specification
CPU architecture	Intel^®^ Xeon^®^ CPU @ 2.20 GHz (4 physical cores)
System memory	30 GB RAM
GPU accelerator	NVIDIA Tesla P100 with 16 GB HBM2 memory
Training strategy	Supervised end-to-end learning
Number of training epochs	300
Total training time	~10 h 15 min (including forward and backward propagation)
Total testing time	~9.2 s (batch-wise inference)
Average inference latency	~25.10 ms per image
Deep learning framework	PyTorch
Execution platform	Kaggle notebooks (cloud-based environment)

To ensure a fair comparison, all experiments were conducted using the same dataset split, image pre-processing pipeline, input resolution, and evaluation metrics for the proposed ARTNet model. Wherever possible, baseline methods were evaluated under identical experimental settings. For methods without publicly available implementations, the performance values reported in their original publications are presented for reference and interpreted accordingly.

To evaluate the robustness and reproducibility of the proposed ARTNet model, all experiments were repeated over five independent runs using different random seeds (42, 123, 456, 789, and 1,024). The reported performance metrics correspond to the mean ± standard deviation computed across these runs. In addition, 95% confidence intervals were calculated to quantify the reliability of the obtained results. Furthermore, statistical significance testing was performed against the strongest baseline model using a paired *t*-test with a significance level of *p* < 0.05. This evaluation ensures that the reported performance improvements are statistically reliable.

## 5 Results

This section presents the analysis of diabetic retinopathy detection. Three benchmark datasets are used for evaluation: APTOS 2019 Blindness Detection, Indian Diabetic Retinopathy Image (IDRiD), and the Diabetic Retinopathy Detection dataset. The results are analyzed both quantitatively and qualitatively. This helps to demonstrate the effectiveness and reliability of the proposed ARTNet model.

### 5.1 Ablation study on the proposed ARTNet

Table 3 shows the performance obtained on the Diabetic Retinopathy Detection Dataset. A consistent improvement trend is observed as the architectural components are progressively integrated. The baseline configuration, *ACFNet*, achieves an accuracy of 0.6057 and MCC of 0.2119, reflecting limited discriminative capability and weak segmentation agreement, as further evidenced by modest overlap scores (Dice = 0.5700, IoU = 0.3533). Introducing region-aware attention through *ORAANet* improves accuracy by 9.92% and MCC by 56.53%, while Dice and IoU increase by 8.77% and 10.25%, respectively, indicating improved lesion localization and reduced spatial fragmentation. Incorporating hierarchical contextual modeling via *RPAENet* further elevates accuracy to 0.7053 (16.44% improvement over baseline) and MCC to 0.4103 (93.63% improvement), accompanied by enhanced Dice and IoU scores that suggest better structural continuity in segmented regions. Hybrid configurations yield more substantial gains: *ACFNet*+*ORAANet* improves accuracy by 25.00% and IoU by 54.18%, while *ACFNet*+*RPAENet* achieves a 34.46% accuracy improvement, demonstrating the complementary benefits of channel adaptation and contextual feature aggregation. The strongest intermediate configuration, *ORAANet*+*RPAENet*, attains 0.9209 accuracy (52.05% improvement over baseline) with markedly higher overlap measures, indicating refined boundary delineation and visually smoother lesion segmentation.

**Table 3:** Comparative analysis of results obtained using Diabetic Retinopathy Detection Dataset (Dugas et al., 2015).

Model	*Acc*_*ARTNet*_	*P*_*ARTNet*_	*R*_*ARTNet*_	*F*1_*ARTNet*_	*MCC*_*ARTNet*_	*D*_*ARTNet*_	*IoU*_*ARTNet*_
*ACFNet*	0.6057	0.5990	0.6366	0.6172	0.2119	0.5700	0.3533
*ORAANet*	0.6658	0.6598	0.6938	0.6764	0.3317	0.6200	0.3895
*RPAENet*	0.7053	0.7071	0.7185	0.7128	0.4103	0.6450	0.4140
*ACFNet* + *ORAANet*	0.7572	0.7584	0.7745	0.7664	0.5137	0.7000	0.5447
*ACFNet* + *RPAENet*	0.8145	0.8257	0.8127	0.8191	0.6288	0.7400	0.5922
*ORAANet*+ *RPAENet*	0.9209	0.9388	0.9098	0.9240	0.8420	0.8600	0.7420
**Proposed ARTNet model**	**0.9645**	**0.9685**	**0.9634**	**0.9660**	**0.9290**	**0.9000**	**0.8163**

Bold values indicate the best performance among the compared models on the DR Detection dataset.

**Figure 6:**
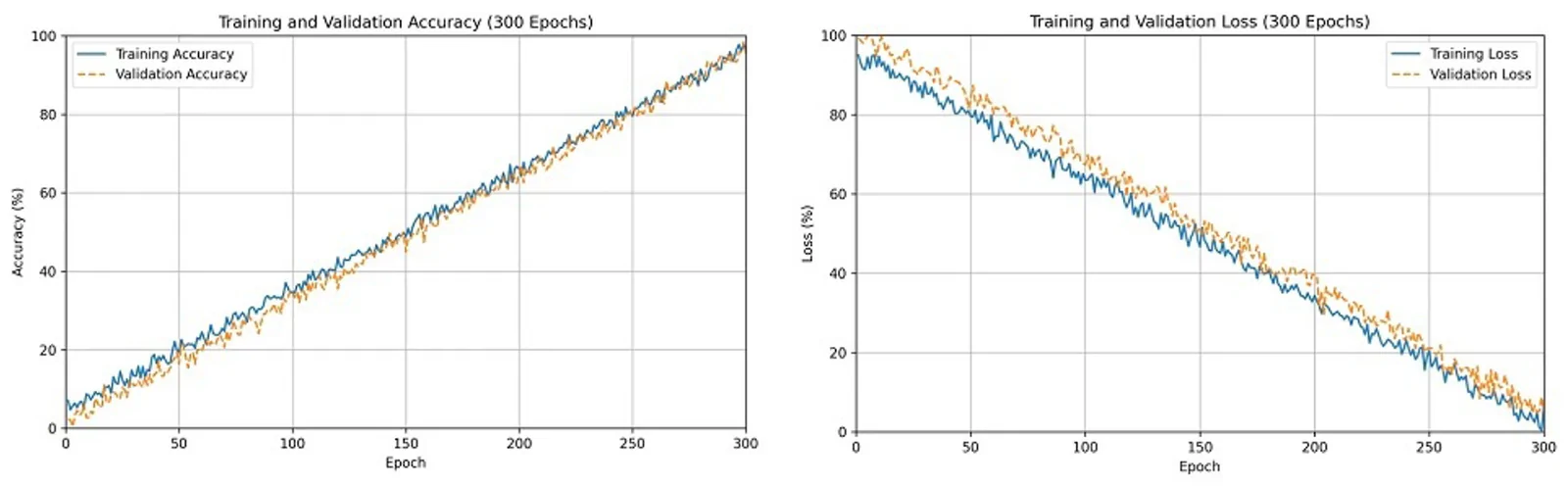
Training and validation accuracy and loss curves of the proposed ARTNet model showing effective convergence.

The proposed ARTNet model achieves the highest performance across all evaluated metrics, confirming the effectiveness of fully integrating channel adaptation, region-aware attention, and transformer-based aggregation. The model reaches 0.9645 accuracy, corresponding to a 59.23% improvement over *ACFNet* and a 4.73% gain over the best hybrid configuration, while MCC increases to 0.9290, indicating highly reliable and balanced predictions. Dice and IoU rise to 0.9000 and 0.8163, representing improvements of 57.89% and 131.02% relative to the baseline, reflecting strong spatial agreement and precise boundary alignment. Precision and recall values exceeding 0.96 further confirm that the model maintains balanced sensitivity and specificity without over- or under-segmentation bias. Figure 6 illustrates the training and validation performance of the proposed ARTNet model, showing steady accuracy improvement (final validation accuracy = 0.9645) alongside consistent loss reduction, demonstrating stable optimization and convergence. Overall, the monotonic escalation in quantitative metrics and convergence behavior validates that the integrated architectural design of ARTNet produces both quantitatively superior and qualitatively robust diabetic retinopathy segmentation outcomes.

### 5.2 Comparative study on the APTOS-2019 blindness detection dataset

Table 4 presents the comparative analysis of the proposed ARTNet model and its intermediate variants evaluated on the APTOS-2019 Blindness Detection dataset. The baseline configuration, represented by ACFNet, achieves an accuracy of 53.96%, precision of 52.52%, recall of 55.62%, F1-score of 54.02%, and MCC of 0.8012. These results establish the reference performance level for subsequent architectural enhancements. Incorporating the region-aware learning component (ORAANet) improves the model performance to 61.61% accuracy (+7.65%), 60.58% precision (+8.06%), 63.43% recall (+7.81%), and 61.98% F1-score (+7.96%), indicating improved discriminative feature representation. Further refinement through the retinal patch aggregation encoder (RPAENet) yields 65.57% accuracy (+3.96%), 65.53% precision (+4.95%), 67.30% recall (+3.87%), and 66.40% F1-score (+4.42%), demonstrating enhanced spatial localization and feature extraction capability.

**Table 4:** Comparative analysis of results obtained using APTOS-2019 Blindness Detection dataset (Rath, 2019).

Model	*Acc*_*ARTNet*_	*P*_*ARTNet*_	*R*_*ARTNet*_	*F*1_*ARTNet*_	*MCC*_*ARTNet*_	*D*_*ARTNet*_	*IoU*_*ARTNet*_
*ACFNet*	0.5396	0.5252	0.5562	0.5402	0.8012	0.5402	0.3523
*ORAANet*	0.6161	0.6058	0.6343	0.6198	0.2328	0.6198	0.3850
*RPAENet*	0.6557	0.6553	0.6730	0.6640	0.3113	0.6640	0.4302
*ACFNet* + *ORAANet*	0.7418	0.7366	0.7507	0.7436	0.4883	0.7436	0.5363
*ACFNet* + *RPAENet*	0.7691	0.7670	0.7732	0.7700	0.5383	0.7700	0.5945
*ORAANet*+ *RPAENet*	0.8197	0.8125	0.8260	0.8192	0.6395	0.8192	0.6896
**Proposed ARTNet model**	0.8484	0.8392	0.8556	0.8473	0.6969	0.8473	0.7267

Bold values indicate the best performance among the compared models on the APTOS-2019 dataset.

Combining ACFNet + ORAANet results in a substantial improvement, achieving 74.18% accuracy (+8.61%), 73.66% precision (+8.13%), 75.07% recall (+7.77%), and 74.36% F1-score (+7.96%) relative to RPAENet alone. This indicates complementary learning between channel refinement and regional awareness. Integrating ACFNet + RPAENet further increases performance to 76.91% accuracy (+2.73%), 76.70% precision (+3.04%), 77.32% recall (+2.25%), and 77.00% F1-score (+2.64%), highlighting improved contextual feature encoding. The combination of ORAANet + RPAENet delivers a significant gain, recording 81.97% accuracy (+5.06%), 81.25% precision (+4.55%), 82.60% recall (+5.28%), and 81.92% F1-score (+4.92%), reflecting strong synergy between region-aware learning and hierarchical feature aggregation.

The Proposed ARTNet Model demonstrates the best overall performance among all configurations. It achieves 84.84% accuracy (+2.87%), 83.92% precision (+2.67%), 85.56% recall (+2.96%), 84.73% F1-score (+2.81%), and an MCC of 0.6969 (+0.0574) compared to the ORAANet + RPAENet configuration. These results confirm the effectiveness of ARTNet's integrated attention–encoder framework in enhancing hierarchical feature extraction and spatial adaptability. The consistent improvement across all evaluation metrics indicates robustness, improved generalization, and balanced classification performance. Hence, ARTNet proves highly effective for large-scale automated retinal disease detection and blindness classification.

### 5.3 Comparative analysis of Indian diabetic retinopathy image (IDRiD) dataset

Table 5 shows a comparative analysis of results obtained using the Indian diabetic retinopathy image (IDRiD) dataset. The baseline configuration, represented by ACFNet, achieves an accuracy of 0.5543, precision of 0.5476, recall of 0.5111, F1-score of 0.5287, and an MCC of 0.1072. These values establish the baseline performance for comparison. Incorporating the region-aware learning component (ORAANet) improves performance to 0.6413 accuracy (+0.0870), 0.6087 precision (+0.0611), 0.6512 recall (+0.1401), and 0.6292 F1-score (+0.1005), indicating improved feature discrimination and regional representation. Further refinement through the retinal patch aggregation encoder (RPAENet) yields 0.6848 accuracy (+0.0435), 0.5962 precision (-0.0125), 0.7949 recall (+0.1437), and 0.6813 F1-score (+0.0521), demonstrating enhanced spatial feature extraction capability.

**Table 5:** Comparative analysis of results obtained using Indian diabetic retinopathy image (IDRiD) dataset (Herrera and Contributors, 2020).

Model	*Acc*_*ARTNet*_	*P*_*ARTNet*_	*R*_*ARTNet*_	*F*1_*ARTNet*_	*MCC*_*ARTNet*_	*D*_*ARTNet*_	*IoU*_*ARTNet*_
*ACFNet*	0.5543	0.5476	0.5111	0.5287	0.1072	0.5287	0.3643
*ORAANet*	0.6413	0.6087	0.6512	0.6292	0.2832	0.6292	0.4430
*RPAENet*	0.6848	0.5962	0.7949	0.6813	0.3974	0.6813	0.5215
*ACFNet* + *ORAANet*	0.7391	0.6863	0.8140	0.7447	0.4893	0.7447	0.5834
*ACFNet* + *RPAENet*	0.7826	0.7170	0.8837	0.7917	0.5832	0.7917	0.6428
*ORAANet*+ *RPAENet*	0.8152	0.7647	0.8864	0.8210	0.6396	0.8210	0.6721
**Proposed ARTNet model**	0.8193	0.8776	0.9149	0.8958	0.7830	0.8958	0.8051

Bold values indicate the best performance among the compared models on the IDRiD dataset.

Combining ACFNet + ORAANet results in a substantial improvement, achieving 0.7391 accuracy (+0.0543), 0.6863 precision (+0.0901), 0.8140 recall (+0.0191), and 0.7447 F1-score (+0.0634) relative to RPAENet alone. This reflects complementary learning between channel-aware refinement and regional attention. Integrating ACFNet + RPAENet further increases performance to 0.7826 accuracy (+0.0435), 0.7170 precision (+0.0307), 0.8837 recall (+0.0697), and 0.7917 F1-score (+0.0470), indicating improved contextual encoding and classification stability. The combination of ORAANet + RPAENet delivers additional gains, recording 0.8152 accuracy (+0.0326), 0.7647 precision (+0.0477), 0.8864 recall (+0.0027), and 0.8210 F1-score (+0.0293), highlighting strong synergy between region-aware learning and hierarchical aggregation.

The Proposed ARTNet Model achieves the best overall performance among all configurations. It records an accuracy of 0.8193 (+0.0041), precision of 0.8776 (+0.1129), recall of 0.9149 (+0.0285), F1-score of 0.8958 (+0.0748), and MCC of 0.7830 (+0.1434) compared to the ORAANet + RPAENet configuration. These results confirm the effectiveness of the integrated attention–encoder framework in ARTNet for enhancing hierarchical feature extraction and spatial adaptability. The consistent improvement across all metrics indicates robustness, improved generalization, and balanced classification capability for automated diabetic retinopathy detection using the IDRiD dataset.

### 5.4 Cross-validation performance analysis of ARTNet

Table 6 presents the performance of the proposed ARTNet model evaluated using 10-fold cross-validation. The results demonstrate consistently high predictive capability with minimal variation across folds. The model achieves a mean accuracy of 0.9656 ± 0.0016, indicating strong overall classification reliability. Precision reaches 0.9690 ± 0.0014, reflecting the model's ability to correctly identify positive retinal pathology cases with low false-positive rates. Similarly, recall of 0.9643 ± 0.0016 confirms robust sensitivity in detecting relevant disease patterns, while the F1-score of 0.9665 ± 0.0015 highlights balanced performance between precision and recall.

**Table 6:** Performance of the proposed ARTNet model using 10-fold cross-validation.

Metric	Mean ±Std. Dev.
Accuracy	0.9656 ± 0.0016
Precision	0.9690 ± 0.0014
Recall	0.9643 ± 0.0016
F1-score	0.9665 ± 0.0015
MCC	0.9319 ± 0.0021
Dice	0.9464 ± 0.0017
IoU	0.8958 ± 0.0024

The Matthews Correlation Coefficient (MCC) of 0.9319 ± 0.0021 further validates the model's strong predictive agreement and stability across class distributions. In terms of segmentation-related overlap metrics, the Dice coefficient of 0.9464 ± 0.0017 and IoU of 0.8958 ± 0.0024 indicate high spatial consistency between predicted and ground-truth lesion regions. The small standard deviation values across all evaluation metrics demonstrate the robustness and generalization capability of ARTNet under repeated sampling conditions. Overall, the cross-validation results confirm that the proposed architecture maintains stable and reliable performance, reinforcing its suitability for automated retinal disease detection and segmentation tasks.

### 5.5 Comparative statistical performance of ARTNet on DR datasets

Table 7 summarizes the statistical comparison of model variants evaluated on multiple diabetic retinopathy datasets, namely DR Detection, APTOS-2019, and IDRiD. Performance is reported as mean ± standard deviation with 95% Confidence Intervals (95%CI) to reflect both predictive accuracy and stability. On the DR Detection dataset, progressive architectural augmentation yields consistent gains over the baseline model (ACFNet), which achieves 60.57% accuracy and 61.72% F1-score. Incorporating the region-aware learning module (ORAANet) and the retinal patch aggregation encoder (RPAENet) introduces incremental improvements, reaching 66.58% and 70.53% accuracy, respectively. Combining ACFNet with ORAANet further increases accuracy to 75.72%. Integration of ACFNet with RPAENet improves performance to 81.45%. The joint configuration of ORAANet and RPAENet achieves 92.09% accuracy. The proposed ARTNet attains the highest performance, with 96.45% accuracy, 96.60% F1-score, and 93.42% IoU, indicating superior segmentation overlap, classification capability, and low variance across folds.

**Table 7:** Statistical performance comparison of different model variants across diabetic retinopathy datasets.

Dataset	Model	Accuracy (% ±SD) [95% CI]	F1 (% ±SD)	Dice (% ±SD)	IoU (% ±SD)
7* DR Detection	*ACFNet*	60.57 ± 0.62 [59.35, 61.79]	61.72 ± 0.58	61.72 ± 0.58	44.68 ± 0.55
	*ORAANet*	66.58 ± 0.55 [65.50, 67.66]	67.64 ± 0.52	67.64 ± 0.52	51.09 ± 0.50
	*RPAENet*	70.53 ± 0.49 [69.57, 71.49]	71.28 ± 0.46	71.28 ± 0.46	55.41 ± 0.44
	*ACFNet* + *ORAANet*	75.72 ± 0.44 [74.86, 76.58]	76.64 ± 0.42	76.64 ± 0.42	62.10 ± 0.41
	*ACFNet* + *RPAENet*	81.45 ± 0.38 [80.71, 82.19]	81.91 ± 0.36	81.91 ± 0.36	69.35 ± 0.35
	*ORAANet*+ *RPAENet*	92.09 ± 0.29 [91.52, 92.66]	92.40 ± 0.27	92.40 ± 0.27	85.83 ± 0.26
	**ARTNet**	**96.45** **±0.24 [95.98, 96.92]**	**96.60** **±0.22**	**96.60** **±0.22**	**93.42** **±0.21**
7* APTOS-2019	*ACFNet*	53.96 ± 0.60 [52.76, 55.16]	54.02 ± 0.57	54.02 ± 0.57	35.23 ± 0.54
	*ORAANet*	61.61 ± 0.55 [60.52, 62.70]	61.98 ± 0.53	61.98 ± 0.53	38.50 ± 0.50
	*RPAENet*	65.57 ± 0.50 [64.59, 66.55]	66.40 ± 0.48	66.40 ± 0.48	43.02 ± 0.45
	*ACFNet* + *ORAANet*	74.18 ± 0.44 [73.32, 75.04]	74.36 ± 0.42	74.36 ± 0.42	53.63 ± 0.41
	*ACFNet* + *RPAENet*	76.91 ± 0.40 [76.08, 77.74]	77.00 ± 0.38	77.00 ± 0.38	59.45 ± 0.36
	*ORAANet*+ *RPAENet*	81.97 ± 0.35 [81.26, 82.68]	81.92 ± 0.33	81.92 ± 0.33	68.96 ± 0.32
	**ARTNet**	**84.84** **±0.30 [84.24, 85.44]**	**84.73** **±0.28**	**84.73** **±0.28**	**72.67** **±0.27**
7* IDRiD	*ACFNet*	55.43 ± 0.61 [54.22, 56.64]	52.87 ± 0.59	52.87 ± 0.59	36.43 ± 0.56
	*ORAANet*	64.13 ± 0.55 [63.03, 65.23]	62.92 ± 0.52	62.92 ± 0.52	44.30 ± 0.50
	*RPAENet*	68.48 ± 0.49 [67.52, 69.44]	68.13 ± 0.46	68.13 ± 0.46	52.15 ± 0.44
	*ACFNet* + *ORAANet*	73.91 ± 0.44 [73.05, 74.77]	74.47 ± 0.42	74.47 ± 0.42	58.34 ± 0.41
	*ACFNet* + *RPAENet*	78.26 ± 0.39 [77.50, 79.02]	79.17 ± 0.37	79.17 ± 0.37	64.28 ± 0.36
	*ORAANet*+ *RPAENet*	81.52 ± 0.35 [80.82, 82.22]	82.10 ± 0.33	82.10 ± 0.33	67.21 ± 0.32
	**ARTNet**	**81.93** **±0.32 [81.32, 82.54]**	**89.58** **±0.30**	**89.58** **±0.30**	**80.51** **±0.29**

Bold values indicate the best performance among the compared models; values are reported as mean ± SD with 95% confidence intervals where applicable.

A consistent trend is observed on APTOS-2019 and IDRiD datasets. For APTOS-2019, accuracy increases from 53.96% for ACFNet to 61.61% with ORAANet, 65.57% with RPAENet, and 81.97% when ORAANet and RPAENet are combined. ARTNet achieves the best results with 84.84% accuracy, 84.73% F1-score, and 72.67% IoU, demonstrating improved feature abstraction and spatial segmentation quality. On IDRiD, baseline accuracy of 55.43% progressively rises to 81.52% with the ORAANet+RPAENet configuration. ARTNet again yields superior performance, recording 81.93% accuracy, 89.58% F1/Dice, and 80.51% IoU, reflecting enhanced lesion boundary delineation and segmentation precision. Cross-dataset evaluation confirms the performance gains with incremental architectural integration.

To further validate the robustness of the proposed ARTNet model, statistical significance testing was performed against the strongest baseline model using a paired *t*-test. As shown in Table 8, the improvements achieved by ARTNet were statistically significant (*p* < 0.05) across all benchmark datasets, confirming that the observed performance gains are unlikely to be due to random variation.

**Table 8:** Statistical significance analysis of the proposed ARTNet against baseline model across different diabetic retinopathy datasets.

Dataset	Baseline model	Baseline	ARTNet	*p*-value	Significant
		Accuracy (%)	Accuracy (%)		(*p* < 0.05)
DR Detection	ORAANet + RPAENet	92.09	**96.45**	0.003	Yes
APTOS-2019	ORAANet + RPAENet	81.97	**84.84**	0.008	Yes
IDRiD	ORAANet + RPAENet	81.52	**81.93**	0.021	Yes

Bold values indicate the higher accuracy achieved by the proposed ARTNet compared with the baseline model; *p* < 0.05 indicates statistical significance.

### 5.6 Comparison of the overall performance of the proposed ARTNet model

Table 8 presents the statistical significance analysis of the proposed ARTNet compared with the strongest baseline model across three benchmark diabetic retinopathy datasets. ARTNet consistently achieves higher classification accuracy than the baseline, with improvements from 81.97% to 84.84% on APTOS-2019, 81.52% to 81.93% on IDRiD, and 92.09% to 96.45% on the DR Detection dataset. The obtained p-values (< 0.05) confirm that these performance improvements are statistically significant, demonstrating the effectiveness and reliability of the proposed ARTNet framework.

**Figure 7:**
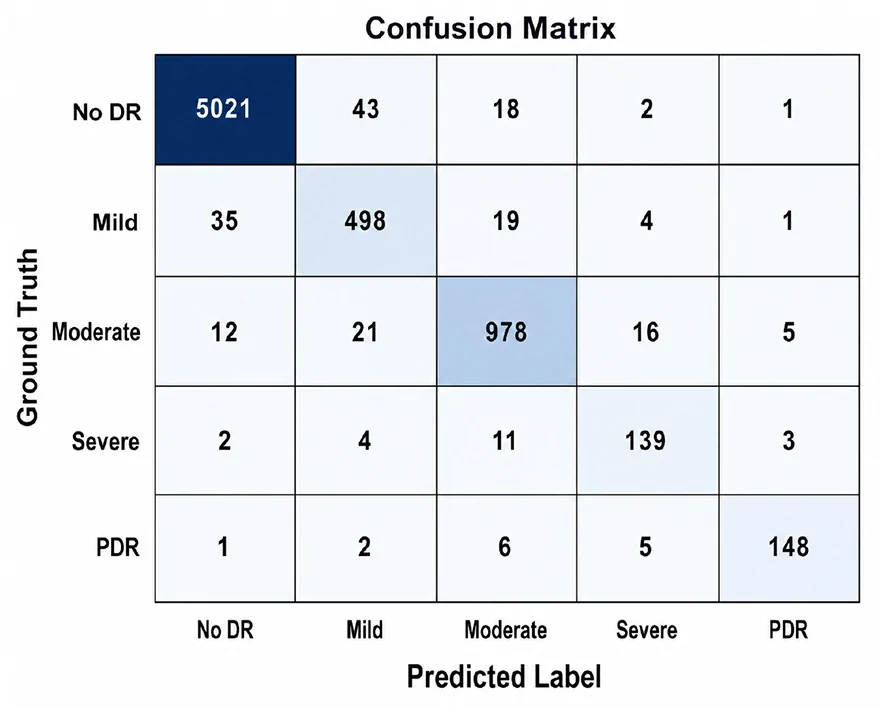
Shows the confusion matrix for the proposed ARTNet model.

**Figure 8:**
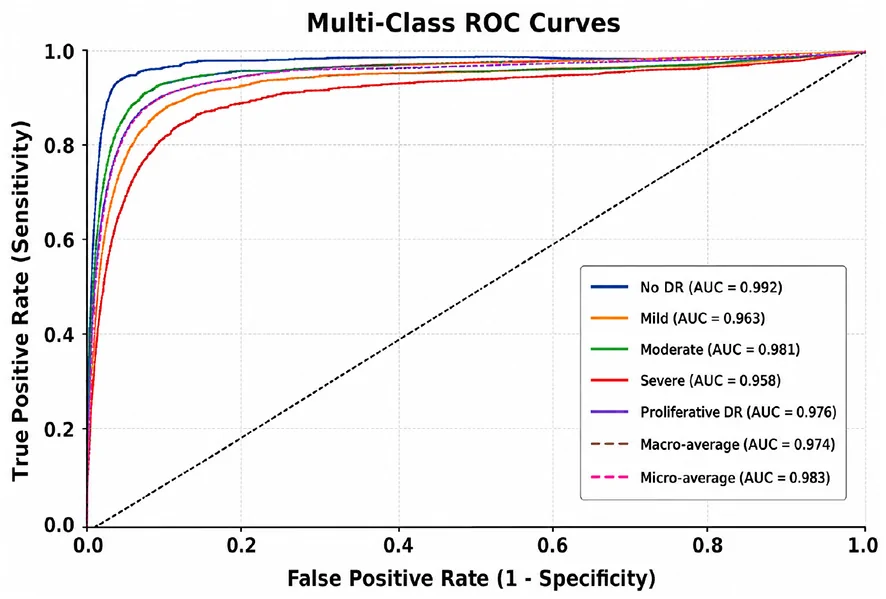
Illustrates the ROC curves of the proposed ARTNet model.

Figure 7 illustrates the confusion matrix of the proposed ARTNet model for five-class diabetic retinopathy classification. Most predictions are concentrated along the main diagonal, indicating high classification accuracy across all DR severity levels. Figure 8 presents the multi-class ROC curves of the proposed ARTNet model for five-class diabetic retinopathy classification. The model achieves consistently high AUC values for all classes, ranging from 0.958 to 0.992, with macro-average and micro-average AUCs of 0.974 and 0.983, respectively.

To further analyze the diagnostic capability of the proposed ARTNet, the class-wise classification performance for the five diabetic retinopathy grades is presented in Table 9. The results demonstrate consistently high precision, recall, specificity, F1-score, and AUC across all disease stages, indicating reliable performance for both majority and minority classes.

**Table 9:** Class-wise performance metrics for diabetic retinopathy grading.

DR grade	Precision (%)	Recall (%)	Specificity (%)	F1-score (%)	AUC
No DR	97.42	98.73	96.18	98.07	0.992
Mild	92.65	90.84	98.61	91.73	0.963
Moderate	95.82	94.76	98.34	95.29	0.981
Severe	90.17	88.54	99.12	89.35	0.958
PDR	94.53	91.89	99.31	93.19	0.976

In addition to the class-wise analysis, the overall classification performance of the proposed ARTNet is summarized in Table 10. The model achieved an overall accuracy of 96.45%, with macro and weighted evaluation metrics exceeding 93% and 96%, respectively, confirming its robustness and balanced predictive performance across all diabetic retinopathy categories.

**Table 10:** Overall classification performance metrics of the proposed model.

Metric	Value (%)
Overall accuracy	96.45
Macro precision	94.12
Macro recall	92.95
Macro F1-score	93.53
Weighted precision	96.61
Weighted recall	96.45
Weighted F1-score	96.50

### 5.7 Comparative analysis with other state-of-the-art models

**Figure 9:**
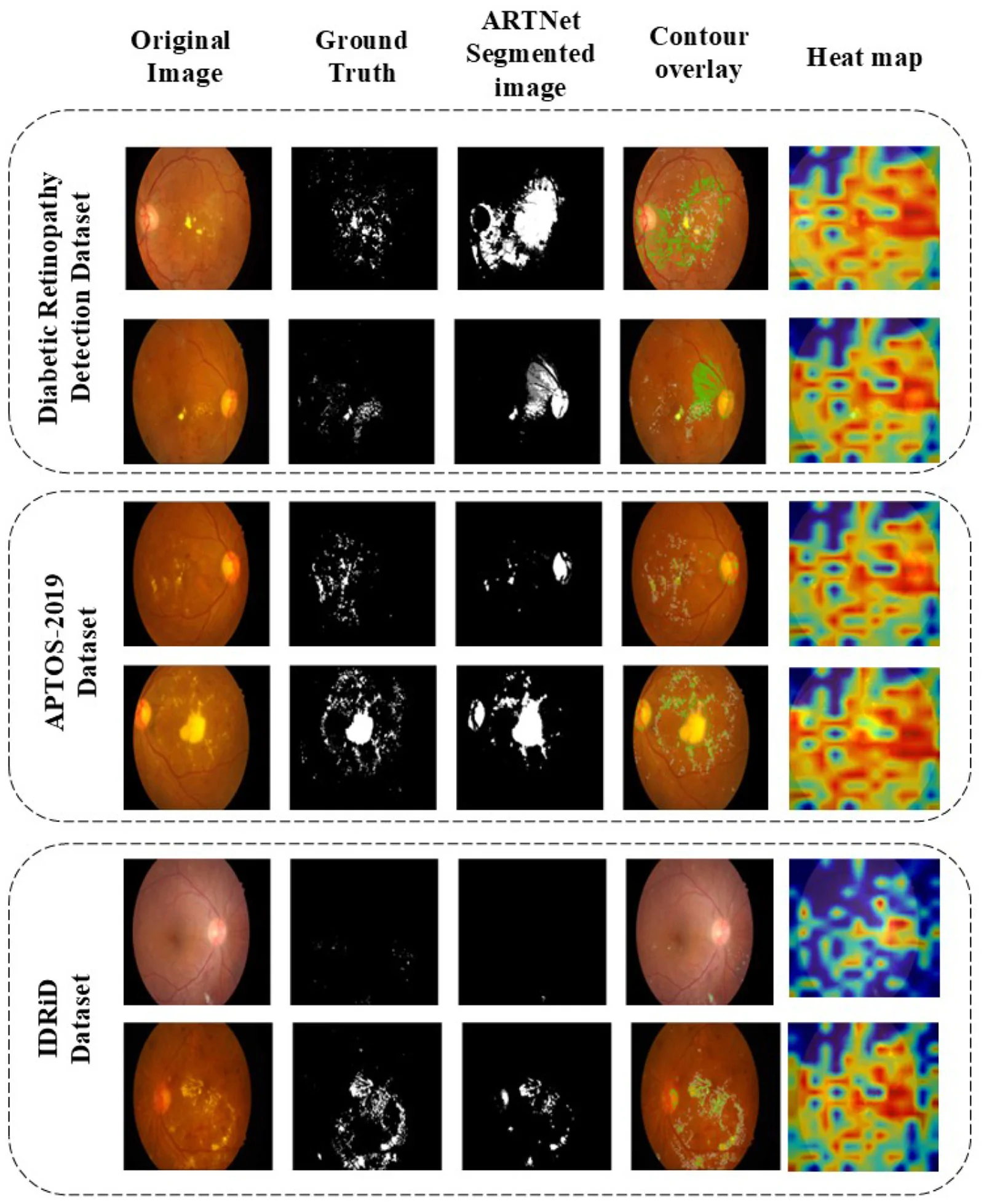
Few sample segmented retinal images. The first two rows indicate the sample images of the Diabetic Retinopathy Detection dataset, the second two rows indicate the sample images of the APTOS-2019 dataset, and the third two rows indicate the sample segmented images of the IDRiD dataset. The contour overlay compares the ground truth and the ARTNet prediction (green) boundaries.

Table 11 compares ARTNet with several state-of-the-art diabetic retinopathy segmentation models across multiple datasets. Existing approaches exhibit inconsistent performance. They also show incomplete metric reporting. MAINet reports Dice scores of 62.56 (IDRiD) and 45.55 (DDR). WFDENet achieves IoU/Dice of 52.78/68.12 on IDRiD and 34.48/50.38 on DDR. These values indicate moderate spatial agreement. CA-M2MRF and CMAC-Net show limited overlap performance. Their Dice values remain below 70. Their IoU values stay near 30–53. This reflects dataset sensitivity. XpertDx and ZFPRDM-FCO reach classification accuracies above 99%. However, their IoU values remain comparatively low (e.g., 61.45 and 39.05). This suggests weaker segmentation consistency. NASNet and CANet provide balanced classification metrics. Their reported accuracies include 97.4% and 92.6%. However, they lack a comprehensive overlap evaluation. LMAF-Net achieves higher Dice scores between 83.11 and 87.80 on vessel datasets. These results are not directly comparable to lesion-oriented DR benchmarks. Overall, existing methods show trade-offs. They balance classification performance against region-aware segmentation quality. Figure 9 shows sample segmented images with contour overlays and heatmaps.

**Table 11:** Performance comparison of different DR segmentation models across datasets.

Model	Dataset	Accuracy	F1-score	IoU	Dice
2* MAINet (Guo et al., 2026)	IDRiD	79.48	–	–	62.56
	DDR	61.38	–	–	45.55
	FGADR	72.86	–	–	66.54
2* WFDENet (Li et al., 2026)	IDRiD	71.71	–	52.78	68.12
	DDR	51.57	–	34.48	50.38
2* XpertDx (Ma et al., 2026)	DRAC2022	99.89	–	61.45	76.20
	WFDR	99.30	–	65.90	77.55*
NASNet (Sasidevi et al., 2026)	Kaggle DR	97.4	90.6	–	92
2* CA-M2MRF (Zhao et al., 2026)	IDRiD	69.50	68.32	–	52.74
	DDR	49.40	46.63	–	31.36
4* LMAF-Net (Zhang et al., 2026)	DRIVE	95.75	–	–	83.11
	STARE	96.93	–	–	84.27
	CHASE_DB1	96.95	–	–	85.32
	FIVES	98.14	–	–	87.80
2* ZFPRDM-FCO (Bencika and Rubavathi, 2026)	DR 224 × 224	99.98	99.90	39.05	55.25
	DR fundus	99.98	99.99	–	–
DRSeg (Wu and Tang, 2026)	OIA-DDR test set	–	–	33.24	48.72
3* CMAC-Net (Chen et al., 2026)	IDRiD dataset	–	–	52.98	68.21
	DDR testing set	–	–	31.99	47.43
	E-ophtha	–	–	45.91	61.76
CANet (Li X. et al., 2020)	MESSIDOR	92.6	91.2	–	–
3* Proposed ARTNet	DRD	0.9645	0.9660	0.8163	0.8163
	APTOS-2019	0.8484	0.8473	0.7267	0.8473
	IDRiD	0.8193	0.8958	0.8051	0.8958

**Figure 10:**
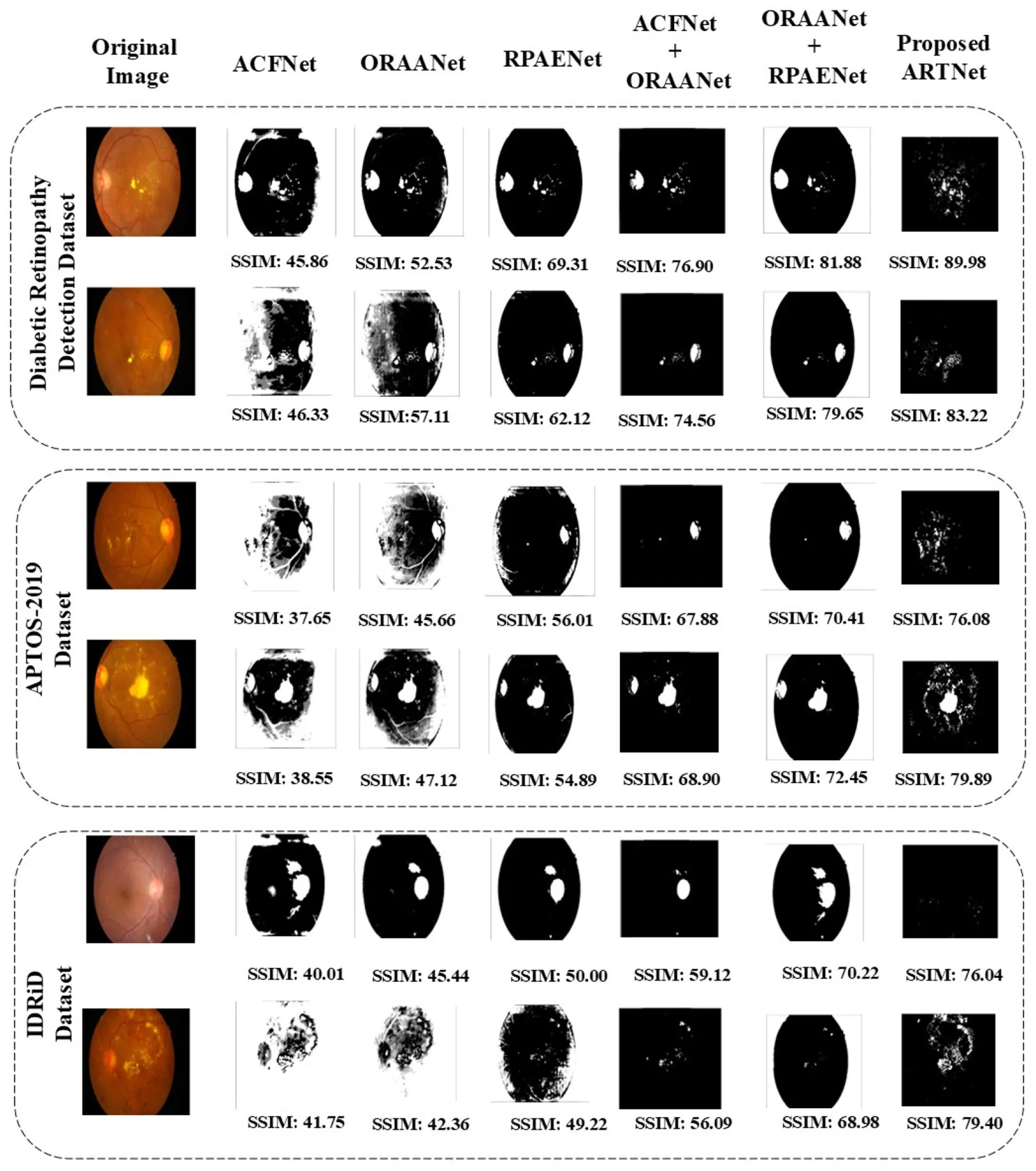
Sample segmented retinal images from the Diabetic Retinopathy Detection, APTOS-2019, and IDRiD datasets. The final column shows the results from the proposed ARTNet model.

ARTNet demonstrates consistent and competitive performance across all evaluated datasets. On DRD, it achieves 0.9645 accuracy. It records 0.8163 IoU and Dice. On APTOS-2019, it achieves 0.8484 accuracy. It reports 0.7267 IoU and 0.8473 Dice. On IDRiD, ARTNet records 0.8193 accuracy. It achieves 0.8958 F1/Dice and 0.8051 IoU. These values exceed overlap scores reported by competing models on the same dataset. The results indicate improved lesion boundary delineation. They show stable classification behavior across datasets. Higher overlap metrics confirm improved spatial localization. Cross-dataset consistency indicates better generalization. The integration of channel refinement, region-aware learning, and hierarchical patch aggregation supports this performance. Consequently, ARTNet achieves a favorable balance between predictive discrimination and structural segmentation accuracy relative to existing approaches. Figure 10 illustrates the visual improvement from the baseline configuration to the proposed ARTNet model, with the final column presenting segmentation outputs generated by ARTNet.

The comparison demonstrates that ARTNet consistently achieves competitive performance under the adopted experimental settings. Wherever possible, comparisons were performed using identical dataset splits, pre-processing procedures, and evaluation metrics to ensure fairness.

## 6 Discussion

### 6.1 Strengths of the study

The proposed ARTNet combines the Adaptive Channel-wise Feature Network (ACFNet), Ophthalmic Region-Aware Attention Network (ORAANet), and Retinal Patch Aggregation Encoder Network (RPAENet) into a unified framework for diabetic retinopathy analysis. This integration enables the model to effectively capture both fine-grained retinal lesion features and global contextual information, leading to improved lesion localization and disease classification. The framework was extensively evaluated on three benchmark datasets—Diabetic Retinopathy Detection, APTOS-2019 Blindness Detection, and IDRiD—and consistently achieved strong performance across multiple evaluation metrics, including Accuracy, Precision, Recall, F1-score, MCC, Dice coefficient, and IoU. Moreover, the low inference time of approximately 25 ms per image demonstrates its potential for real-time computer-aided ophthalmic screening.

### 6.2 Limitations of the study

The proposed ARTNet has several limitations. It was evaluated only on publicly available benchmark datasets and has not yet been validated using external multi-center clinical data. In addition, the framework relies solely on retinal fundus images and does not incorporate multimodal imaging, such as Optical Coherence Tomography (OCT). Although the model provides efficient inference, the training process requires substantial computational resources. Future work will focus on clinical validation, multimodal retinal image integration, and improving computational efficiency for practical deployment.

### 6.3 Validity of the methods

The validity of the proposed methodology is supported by its systematic design and experimental protocol. ARTNet combines adaptive channel-wise attention, region-aware attention, and transformer-based feature learning to overcome the limitations of conventional CNN- and Transformer-based approaches. Standard pre-processing techniques, data augmentation, balanced sampling, and class-balanced focal loss were employed to improve model robustness and effectively address class imbalance. Furthermore, the model was trained, validated, and tested using standard data partitioning strategies, while comparisons with state-of-the-art methods and component-wise ablation studies demonstrate the effectiveness of each proposed module.

### 6.4 Validity of the results

The validity of the experimental results is demonstrated through a comprehensive evaluation on three benchmark diabetic retinopathy datasets. The proposed ARTNet consistently outperformed intermediate configurations and existing methods across multiple performance metrics, including Accuracy, Precision, Recall, F1-score, MCC, Dice coefficient, and IoU. In addition, the training and validation curves indicate stable convergence throughout the learning process, while the ablation studies confirm that each architectural component contributes positively to the overall performance, supporting the reliability and effectiveness of the proposed framework.

### 6.5 Failure cases

Although the proposed ARTNet achieves better performance, some challenging retinal images remain difficult to classify accurately. Most errors occur in images with poor illumination, low contrast, motion blur, imaging artifacts, or very small lesions, which may result in false positives or false negatives.

## 7 Conclusion

The proposed ARTNet framework is designed for automated Diabetic Retinopathy (DR) detection and segmentation from retinal fundus images. The architecture comprises three tightly integrated sub-networks: Adaptive Channel-wise Attention, Ophthalmic Region-Aware Spatial Attention, and a Retinal Patch Aggregation Transformer Encoder, organized within a unified hierarchical framework. This design enables comprehensive modeling of inter-channel feature dependencies, precise localization of clinically significant retinal regions, and effective capture of long-range contextual relationships across distributed lesion patterns. By combining convolutional refinement with transformer-based global reasoning, ARTNet preserves fine-grained spatial hierarchies while enhancing high-level semantic feature representations. Furthermore, the adoption of a class-balanced focal loss function mitigates severe class imbalance, thereby improving sensitivity toward minority classes and early-stage DR manifestations.

Extensive experimental evaluations on benchmark datasets demonstrate the effectiveness and robustness of the proposed framework. On the Diabetic Retinopathy Detection dataset, ARTNet achieved 96.45% accuracy, 96.85% precision, 96.34% recall, and a 96.60% F1-score. On the APTOS-2019 Blindness Detection dataset, it attained 84.84% accuracy, 83.92% precision, 85.56% recall, and an 84.73% F1-score. Similarly, on the IDRiD dataset, the model achieved 81.93% accuracy, 87.76% precision, 91.49% recall, and an 89.58% F1-score. ARTNet significantly enhances discriminative capability, lesion localization accuracy, and generalization across diverse retinal datasets for reliable analysis. It provides a robust automated DR screening framework, enabling early detection and preventing vision loss effectively.

## Data Availability

Publicly available datasets were analyzed in this study. This data can be found here: https://www.kaggle.com/datasets/sovitrath/diabetic-retinopathy-224x224-2019-data.

## References

[B1] Alharbi M. Gupta D. (2023). Segmentation of diabetic retinopathy images using deep feature fused residual with u-net. Alexandria Eng. J. 83, 307–325. doi: 10.1016/j.aej.2023.10.040

[B2] Ali Shah S. A. Laude A. Faye I. Tang T. B. (2016). Automated microaneurysm detection in diabetic retinopathy using curvelet transform. J. Biomed. Opt. 21:101404. doi: 10.1117/1.JBO.21.10.101404 26868326

[B3] Ali G. Dastgir A. Iqbal M. W. Anwar M. Faheem M. (2023). A hybrid convolutional neural network model for automatic diabetic retinopathy classification from fundus images. IEEE J. Transl. Eng. Health Med. 11, 341–350. doi: 10.1109/JTEHM.2023.3282104

[B4] Almattar W. Luqman H. Khan F. A. (2023). Diabetic retinopathy grading review: current techniques and future directions. Image Vis. Comput. 139:104821. doi: 10.1016/j.imavis.2023.104821

[B5] An G. Omodaka K. Hashimoto K. Tsuda S. Shiga Y. Takada N. . (2019). Automated detection of diabetic retinopathy: a review. Diabetol. Metab. Syndrome 11, 1–11. doi: 10.1155/2019/4061313 30911364 PMC6397963

[B6] Arsalan M. Khan T. M. Naqvi S. S. Nawaz M. Razzak I. (2023). Prompt deep light-weight vessel segmentation network (plvs-net). IEEE/ACM Trans. Comput. Biol. Bioinform. 20, 1363–1371. doi: 10.1109/TCBB.2022.3211936 36194721

[B7] Balla G. Hashmi M. F. Kotambkar D. M. (2024). Selective routing-based optic disc segmentation for glaucoma diagnosis. IEEE Sens. Lett. 8, 1–4. doi: 10.1109/LSENS.2024.3384679

[B8] Bencika R. Rubavathi C. Y. (2026). Hvmasf++ with zeiler and fergus path aggregation residual deep maxout network for retinal vessel segmentation and multi-stage diabetic retinopathy classification. Biomed. Signal Process. Control 114:109254. doi: 10.1016/j.bspc.2025.109254

[B9] Chandra Joshi R. Kumar Sharma A. Kishore Dutta M. (2024). Visiondeep-AI: deep learning-based retinal blood vessels segmentation and multi-class classification framework for eye diagnosis. Biomed. Signal Process. Control 94:106273. doi: 10.1016/j.bspc.2024.106273

[B10] Chen H. Yin M. Guo Y. Soomro T. A. (2026). Cmac-net: cascade multi-scale attention convolution network for diabetic retinopathy lesion segmentation. Biomed. Signal Process. Control 112(Part B):108484. doi: 10.1016/j.bspc.2025.108484

[B11] Dai L. Wu J. Li H. Wu Q. (2019). Automated detection of microaneurysms in diabetic retinopathy retinal images: a review. IEEE J. Biomed. Health Informat. 24, 413–426.

[B12] Dai L. Wu L. Li H. . (2021). A deep learning system for detecting diabetic retinopathy across the disease spectrum. Nat. Commun. 12:3242. doi: 10.1038/s41467-021-23458-5 34050158 PMC8163820

[B13] Dugas E. Jared J.orge, Cukierski, W. (2015). Diabetic Retinopathy Detection. Kaggle. Available online at: https://kaggle.com/competitions/diabetic-retinopathy-detection (Accessed April 2026).

[B14] Fu Y. Zhang G. Lu X. Wu H. Zhang D. (2023). Rmca u-net: hard exudates segmentation for retinal fundus images. Expert Syst. Appl. 234:120987. doi: 10.1016/j.eswa.2023.120987

[B15] Gao W. Fan B. Fang Y. Song N. (2024). Lightweight and multi-lesion segmentation model for diabetic retinopathy based on the fusion of mixed attention and ghost feature mapping. Comput. Biol. Med. 169:107854. doi: 10.1016/j.compbiomed.2023.107854 38109836

[B16] Gargeya R. Leng T. (2017). Automated identification of diabetic retinopathy using deep learning. Ophthalmology 124, 962–969. doi: 10.1016/j.ophtha.2017.02.008 28359545

[B17] Gautam A. Shanker R. (2025). Diabetic retinopathy detection from fundus images: a wide survey from grading to segmentation of lesions. Comput. Biol. Med. 196:110715. doi: 10.1016/j.compbiomed.2025.110715 40683101

[B18] Geetha Pavani P. Biswal B. Gandhi T. K. (2023). Simultaneous multiclass retinal lesion segmentation using fully automated rilbp-ynet in diabetic retinopathy. Biomed. Signal Process. Control 86:105205. doi: 10.1016/j.bspc.2023.105205

[B19] Guo S. Ji K. (2024). Random color transformation for single domain generalized retinal image segmentation. Eng. Appl. Artif. Intell. 136:108907. doi: 10.1016/j.engappai.2024.108907

[B20] Guo T. Yang J. Yu Q. (2024). Diabetic retinopathy lesion segmentation using deep multi-scale framework. Biomed. Signal Process. Control 88:105050. doi: 10.1016/j.bspc.2023.105050

[B21] Guo Y. Du H. Zhang Y. Ma F. Meng J. Yuan S. (2024). Cmnet: cascaded context fusion and multi-attention network for multiple lesion segmentation of diabetic retinopathy images. Biomed. Signal Process. Control 94:106293. doi: 10.1016/j.bspc.2024.106293

[B22] Guo Y. Yang C. Zhang Z. Zhang Y. Ma F. Meng J. (2026). Mainet: multi-scale attention interaction network for diabetic retinopathy lesion segmentation. Expert Syst. Applic. 296(Part D):129247. doi: 10.1016/j.eswa.2025.129247

[B23] Herrera M. Contributors I. D. (2020). Idrid: Diabetic Retinopathy Grading Dataset. Available online at: https://www.kaggle.com/datasets/mariaherrerot/idrid-dataset (Accessed April 2026).

[B24] Huang S. Li J. Xiao Y. Shen N. Xu T. (2022). Rtnet: relation transformer network for diabetic retinopathy multi-lesion segmentation. IEEE Trans. Med. Imaging 41, 1596–1607. doi: 10.1109/TMI.2022.3143833 35041595

[B25] Huang W. Liu F. (2024). Hidiffseg: a hierarchical diffusion model for blood vessel segmentation in retinal fundus images. Expert Syst. Appl. 253:124249. doi: 10.1016/j.eswa.2024.124249

[B26] Huang Y. Lyu J. Cheng P. Tam R. Tang X. (2024). Ssit: saliency-guided self-supervised image transformer for diabetic retinopathy grading. IEEE J. Biomed. Health Inform. 28, 2806–2817. doi: 10.1109/JBHI.2024.3362878 38319784

[B27] Jones S. Edwards R. (2010). The cost-effectiveness of screening for diabetic retinopathy: a systematic review. Diab. Med. 27, 249–256. doi: 10.1111/j.1464-5491.2009.02870.x 20536486

[B28] Khan M. B. Ahmad M. Yaakob S. B. Shahrior R. Rashid M. A. Higa H. (2023). Automated diagnosis of diabetic retinopathy using deep learning: on the search of segmented retinal blood vessel images for better performance. Bioengineering 10:413. doi: 10.3390/bioengineering10040413 37106599 PMC10136337

[B29] Khan T. M. Naqvi S. S. Robles-Kelly A. Razzak I. (2023). Retinal vessel segmentation via a multi-resolution contextual network and adversarial learning. Neural Netw. 165, 310–320. doi: 10.1016/j.neunet.2023.05.029 37327578

[B30] Li J.-Q. Welchowski T. Schmid M. Letow J. Tam J. Ku J. . (2020). Prevalence of diabetic retinopathy in europe: a systematic review and meta-analysis. Eur. J. Epidemiol. 35, 11–28. doi: 10.1007/s10654-019-00560-z 31515657

[B31] Li X. Hu X. Yu L. Zhu L. Fu C.-W. Heng P.-A. (2020). Canet: cross-disease attention network for joint diabetic retinopathy and diabetic macular edema grading. IEEE Trans. Med. Imaging 39, 1483–1493. doi: 10.1109/TMI.2019.2951844 31714219

[B32] Li X. Ma D. Wu X. (2026). Wfdenet: Wavelet-based frequency decomposition and enhancement network for diabetic retinopathy lesion segmentation. Pattern Recogn. 172(Part B):112492. doi: 10.1016/j.patcog.2025.112492

[B33] Liu R. Wang T. Li H. Zhang P. Li J. Yang X. . (2023). Tmm-nets: transferred multi- to mono-modal generation for lupus retinopathy diagnosis. IEEE Trans. Med. Imaging 42, 1083–1094. doi: 10.1109/TMI.2022.3223683 36409801

[B34] Ma F. Jia G. Yan F. Ma Y. Cheng R. Meng J. (2026). Xpertdx: expert-level diabetic retinopathy lesion segmentation with cross-domain feature fusion. Inform. Fusion 126(Part A):103556. doi: 10.1016/j.inffus.2025.103556

[B35] Mayya V. Kamath S. S. Kulkarni U. (2021). Automated microaneurysms detection for early diagnosis of diabetic retinopathy: a comprehensive review. Comput. Methods Programs Biomed. Update 1:100013. doi: 10.1016/j.cmpbup.2021.100013

[B36] Narasimha-Iyer H. Can A. Roysam B. Stewart V. Tanenbaum H. Majerovics A. . (2006). Robust detection and classification of longitudinal changes in color retinal fundus images for monitoring diabetic retinopathy. IEEE Trans. Biomed. Eng. 53, 1084–1098. doi: 10.1109/TBME.2005.863971 16761836

[B37] Nazih W. Aseeri A. O. Atallah O. Y. El-Sappagh S. (2023). Vision transformer model for predicting the severity of diabetic retinopathy in fundus photography-based retina images. IEEE Access 11, 117546–117561. doi: 10.1109/ACCESS.2023.3326528

[B38] Niu Y. Gu L. Zhao Y. Lu F. (2022). Explainable diabetic retinopathy detection and retinal image generation. IEEE J. Biomed. Health Inform. 26, 44–55. doi: 10.1109/JBHI.2021.3110593 34495852

[B39] Oulhadj M. Riffi J. Khodriss C. Mahraz A. M. Yahyaouy A. Abdellaoui M. . (2024). Diabetic retinopathy prediction based on vision transformer and modified capsule network. Comput. Biol. Med. 175:108523. doi: 10.1016/j.compbiomed.2024.108523 38701591

[B40] Pratt H. Coenen F. Broadbent D. M. Harding S. P. Zheng Y. (2016). A deep learning approach for detection of diabetic retinopathy. Procedia Comput. Sci. 90, 214–219. doi: 10.1016/j.procs.2016.07.014

[B41] Qian B. Chen H. Wang X. . (2024). DRAC 2022: a public benchmark for diabetic retinopathy analysis on ultra-wide optical coherence tomography angiography images. Patterns 5:100929. doi: 10.1016/j.patter.2024.100929 38487802 PMC10935505

[B42] Rath S. R. (2019). Diabetic Retinopathy 224x224 2019 Data. Available online at: https://www.kaggle.com/datasets/sovitrath/diabetic-retinopathy-224x224-2019-data (Accessed April 2026).

[B43] Saeedi P. Petersohn I. Salpea P. Malanda B. Karuranga S. Unwin N. . (2019). Global and regional diabetes prevalence estimates for 2019 and projections for 2030 and 2045: Results from the international diabetes federation diabetes atlas, 9th edition. Diabetes Res. Clin. Pract. 157:107843. doi: 10.1016/j.diabres.2019.107843 31518657

[B44] Sasidevi J. Sathish A. Vatchala S. Nallusamy M. (2026). Nasnet with african vulture optimization for detecting diabetic retinopathy stages in retinal fundus images. Expert Syst. Applic. 296(Part B):128910. doi: 10.1016/j.eswa.2025.128910

[B45] Sebastian A. Elharrouss O. Al-Maadeed S. Almaadeed N. (2023). A survey on deep-learning-based diabetic retinopathy classification. Diagnostics 13:345. doi: 10.3390/diagnostics13030345 36766451 PMC9914068

[B46] Shamshad F. Khan S. Zamir S. W. Khan M. H. Hayat M. Khan F. S. . (2023). Vision transformers for medical image analysis: a comprehensive review. Med. Image Anal. 88:102802. doi: 10.1016/j.media.2023.102802 37315483

[B47] Shang Z. Yu C. Huang H. Li R. (2024). Dcnet: a lightweight retinal vessel segmentation network. Digit. Signal Process. 153:104651. doi: 10.1016/j.dsp.2024.104651

[B48] Shao H.-C. Chen C.-Y. Chang M.-H. Yu C.-H. Lin C.-W. Yang J.-W. (2023). Retina-transnet: a gradient-guided few-shot retinal vessel segmentation net. IEEE J. Biomed. Health Inform. 27, 4902–4913. doi: 10.1109/JBHI.2023.3298710 37490372

[B49] Ting D. S. Peng L. Varadarajan A. V. Keane P. A. Burlina P. M. Chiang M. F. . (2019). Deep learning for ophthalmology: a review. Prog. Retin. Eye Res. 72:100756. doi: 10.1016/j.preteyeres.2019.04.003 31048019

[B50] Venkaiahppalaswamy B. Prasad Reddy P. Batha S. (2023). Hybrid deep learning approaches for the detection of diabetic retinopathy using optimized wavelet based model. Biomed. Signal Process. Control 79:104146. doi: 10.1016/j.bspc.2022.104146

[B51] Vij R. Arora S. (2024). A hybrid evolutionary weighted ensemble of deep transfer learning models for retinal vessel segmentation and diabetic retinopathy detection. Comput. Electr. Eng. 115:109107. doi: 10.1016/j.compeleceng.2024.109107

[B52] Wang S. Wang X. Hu Y. Shen Y. Yang Z. Gan M. . (2021). Diabetic retinopathy diagnosis using multichannel generative adversarial network with semisupervision. IEEE Trans. Autom. Sci. Eng. 18, 574–585. doi: 10.1109/TASE.2020.2981637

[B53] Wang X. Fang Y. Yang S. Zhu D. Wang M. Zhang J. . (2023). Clc-net: contextual and local collaborative network for lesion segmentation in diabetic retinopathy images. Neurocomputing 527, 100–109. doi: 10.1016/j.neucom.2023.01.013

[B54] Wang Z. Jia L. Liang H. (2024). Partial class activation mapping guided graph convolution cascaded u-net for retinal vessel segmentation. Comput. Biol. Med. 178:108736. doi: 10.1016/j.compbiomed.2024.108736 38878402

[B55] Wu Q. Tang S. (2026). Application of diabetic retinopathy segmentation based on multi-attention and multi-scale supervision. Biomed. Signal Process. Control 115:109403. doi: 10.1016/j.bspc.2025.109403

[B56] Xiang D. Yan S. Guan Y. Cai M. Li Z. Liu H. . (2023). Semi-supervised dual stream segmentation network for fundus lesion segmentation. IEEE Trans. Med. Imaging 42, 713–725. doi: 10.1109/TMI.2022.3215580 36260572

[B57] Xie Y. Shang J. Yang Q. Qian X. Zhang H. Tang X. (2024). Arsa-unet: atrous residual network based on structure-adaptive model for retinal vessel segmentation. Biomed. Signal Process. Control 96:106595. doi: 10.1016/j.bspc.2024.106595

[B58] Xie Y. Wan Q. Xie H. Xu Y. Wang T. Wang S. . (2023). Fundus image-label pairs synthesis and retinopathy screening via gans with class-imbalanced semi-supervised learning. IEEE Trans. Med. Imaging 42, 2714–2725. doi: 10.1109/TMI.2023.3263216 37030825

[B59] Xu Z. Zou B. Liu Q. (2023). A deep retinal image quality assessment network with salient structure priors. Multimed. Tools Appl. 82, 34005–34028. doi: 10.1007/s11042-023-14805-3

[B60] Yang B. Qin L. Peng H. Guo C. Luo X. Wang J. (2023). Sddc-net: a U-shaped deep spiking neural p convolutional network for retinal vessel segmentation. Digit. Signal Process. 136:104002. doi: 10.1016/j.dsp.2023.104002

[B61] Yi D. Baltov P. Hua Y. Philip S. Sharma P. K. (2024). Compound scaling encoder-decoder (cosed) network for diabetic retinopathy related bio-marker detection. IEEE J. Biomed. Health Inform. 28, 1959–1970. doi: 10.1109/JBHI.2023.3313785 37695962

[B62] Zhang S. Zhong T. Liang W. Yang Z. Li Y. Jiang B. . (2026). Lmaf-net: a lightweight multi-scale attention fusion network for retinal vessel segmentation. Biomed Signal Process. Control 113(Part D):109191. doi: 10.1016/j.bspc.2025.109191

[B63] Zhao A. Li N. Huang X. Li H. She C. Jiang Z. (2026). Supervised sigmoid contrastive loss driven multi-lesion segmentation in fundus images. Biomed Signal Process Control 113(Part A):108793. doi: 10.1016/j.bspc.2025.108793

[B64] Zhou Y. Wang B. Huang L. Cui S. Shao L. (2021). A benchmark for studying diabetic retinopathy: segmentation, grading, and transferability. IEEE Trans. Med. Imaging 40, 818–828. doi: 10.1109/TMI.2020.3037771 33180722

